# Loss of Heterozygosity Drives Adaptation in Hybrid Yeast

**DOI:** 10.1093/molbev/msx098

**Published:** 2017-03-29

**Authors:** Caiti S. Smukowski Heil, Christopher G. DeSevo, Dave A. Pai, Cheryl M. Tucker, Margaret L. Hoang, Maitreya J. Dunham

**Affiliations:** 1 Department of Genome Sciences, University of Washington, Seattle, WA; 2 Lewis-Sigler Institute for Integrative Genomics, Princeton University, Princeton, NJ; 3 Department of Embryology, Howard Hughes Medical Institute, Carnegie Institution, Baltimore, MD; 4 Department of Biology, Johns Hopkins University, Baltimore, MD

**Keywords:** hybrid, adaptation, loss of heterozygosity, experimental evolution, *Saccharomyces uvarum*, *Saccharomyces cerevisiae*

## Abstract

Hybridization is often considered maladaptive, but sometimes hybrids can invade new ecological niches and adapt to novel or stressful environments better than their parents. The genomic changes that occur following hybridization that facilitate genome resolution and/or adaptation are not well understood. Here, we examine hybrid genome evolution using experimental evolution of de novo interspecific hybrid yeast *Saccharomyces cerevisiae* × *Saccharomyces uvarum* and their parentals. We evolved these strains in nutrient-limited conditions for hundreds of generations and sequenced the resulting cultures identifying numerous point mutations, copy number changes, and loss of heterozygosity (LOH) events, including species-biased amplification of nutrient transporters. We focused on a particularly interesting example, in which we saw repeated LOH at the high-affinity phosphate transporter gene *PHO84* in both intra- and interspecific hybrids. Using allele replacement methods, we tested the fitness of different alleles in hybrid and *S. cerevisiae* strain backgrounds and found that the LOH is indeed the result of selection on one allele over the other in both *S. cerevisiae* and the hybrids. This is an example where hybrid genome resolution is driven by positive selection on existing heterozygosity and demonstrates that even infrequent outcrossing may have lasting impacts on adaptation.

## Introduction

Hybridization is now recognized as a common phenomenon across the tree of life. Historically however, the detection of hybrids has been difficult, and its incidence may be under-reported for both plants and animals, and almost certainly for certain eukaryotes like insects and fungi (Bullini 1994; Albertin and Marullo 2012). Its importance as an evolutionary force has thus been maligned, as hybrids appeared both rare and typically at a reduced fitness. In addition to potential postreproductive barriers, the hybrid is theorized to be ill-adapted to its environment and will also suffer minority cytotype disadvantage, because other hybrids are uncommon and backcrosses to parental species may be unfit (Mallet 2007). However, hybrids can have a variety of advantages over their parents, including heterozygote advantage, extreme phenotypic traits, and reproductive isolation (usually resulting from polyploidy), and can thus facilitate adaptation to novel or stressful conditions, invade unoccupied ecological niches, and even increase biodiversity.

Some hybridization events lead to new hybrid species (Rieseberg 1997; Nolte et al. 2005; Mavarez et al. 2006; Meyer et al. 2006; Soltis and Soltis 2009; Schumer et al. 2014), whereas most result in introgression from hybrid backcrosses to the more abundant parental species (Dowling et al. 1989; Taylor and Hebert 1993; Wayne 1993; Grant et al. 2005; Dasmahapatra et al. 2012). Hybridization introduces genetic variation into a population at orders of magnitude greater than what mutation alone can achieve, in a sense operating as a “multi-locus macro-mutation” (Grant and Grant 1994; Barton 2001; Mallet 2007; Abbott et al. 2013). Therefore, hybridization via introgression, polyploidy, or homoploid hybrid speciation may offer a rapid strategy for adaptation to changing environmental conditions. For example, in Darwin’s finches, adaptive introgression supplied the morphological variation that allowed the species to survive following an El Niño event (Grant and Grant 2010, 2002), and in ancient humans, introgression allowed adaptation to high altitudes (Huerta-Sanchez and Casey 2015), among other traits (Racimo et al. 2015). The most iconic example comes from the hybrid sunflower species *Helianthus anomalus*, *Helianthus deserticola*, and *Helianthus paradoxus*, from the parents *Helianthus annuus* and *Helianthus petiolaris*. These three hybrid species are locally adapted to extreme desert, salt marsh, and dune habitats, respectively, and show traits such as increased drought or salt tolerance relative to their parents (Heiser 1954; Rieseberg 1991; Schwarzbach et al. 2001; Rosenthal et al. 2002).

Agriculture and industry use both intra- and interspecific hybrids as a tool to increase yield or robustness, introduce resistance to pests, and create novel phenotype or flavor profiles. For example, plant breeders have crossed domesticated species to wild species to introduce resistance to a variety of pathogens in wheat, potato, and canola (Mason and Batley 2015), and almost all maize grown in the United States is grown from intraspecific hybrid seeds, which has increased yield and provided improved resistance to biotic and abiotic factors (Crow 1998). Vintners and brewers have created interspecific hybrids to select for traits such as lower acetic acid concentration (Bellon et al. 2015), and many incidental fungal hybrids have been discovered in brewing and industry, including *Pichia sorbitophila* (Louis et al. 2012), and various hybrids across the *Saccharomyces* clade (Gonzalez et al. 2006, 2008; Muller and McCusker 2009; Hittinger 2013; Bellon et al. 2015), most notably the lager-brewing yeast, *Saccharomyces pastorianus* (Tamai et al. 1998; Dunn and Sherlock 2008; Walther et al. 2014; Baker et al. 2015; Gibson and Liti 2015; Peris et al. 2016). It is presumed that the severe selection pressures exerted during industrial processes have selected for interspecific hybrid genomes that may be more able to cope with the extreme environments.

At the genomic level, hybridization induces chromosome loss/aneuploidy, chromosomal rearrangements, gene loss, changes in gene expression, changes in epigenetic modifications, transposable element mobilization, and large-scale loss of heterozygosity (LOH), in which the allele of one species is lost and the allele of the other species is retained and may even be duplicated via gene conversion or break-induced replication (Masly et al. 2006; Landry et al. 2007; Doyle et al. 2008; Michalak 2009; Ainouche and Jenczewski 2010; Albertin and Marullo 2012; Abbott et al. 2013; Soltis 2013; Borneman et al. 2014; Soltis et al. 2014). These extensive changes can result in a chimeric, stabilized hybrid, although the period of time for genome stabilization to occur can range dramatically (Soltis et al. 2014). It is unknown whether there are structural and functional biases in the ways in which genes/alleles are lost or modified. Both drift and selection influence the resolution of the hybrid genome, but their contributions are difficult to untangle.

Researchers have long been exploring the genetics of hybrid traits in the lab, particularly in agricultural crops, although this is often slowed by infertility and reduced viability in many interspecific hybrids (Perez-Prat and van Lookeren Campagne 2002; Hajjar and Hodgkin 2007; Ouyang et al. 2010). The *Saccharomyces* genus, which includes the budding yeast *Saccharomyces cerevisiae*, lends itself particularly well to experimental study. Many hybrids of this genus have been discovered in brewing, industrial, and natural environments; indeed, the genus itself is speculated to have been founded by the product of an ancient hybridization event (Hittinger 2013; Marcet-Houben and Gabaldon 2015; Barbosa et al. 2016; Leducq et al. 2016). Viable interspecific hybrids can be created de novo in the lab (Marinoni et al. 1999; Greig et al. 2002), and their ability to grow mitotically means that the catastrophic postzygotic barriers to speciation that generally doom other obligate sexually reproducing hybrids can be avoided. This experimental system allows us to observe evolution in real time in the laboratory environment, and the genetic and genomic tools available in this model genus facilitate characterization of the connection between genotype and phenotype, including fitness.

Previous work in our lab group has utilized experimental evolution to investigate adaptive events in haploid and homozygous diploid *S. cerevisiae* (Gresham et al. 2008; Payen et al. 2014; Sunshine et al. 2015). To investigate genome evolution post hybridization, we utilize an interspecific hybrid, *S. cerevisiae* × *Saccharomyces uvarum*, and its parentals: a homozygous diploid *S. uvarum* and an intraspecific hybrid S*. cerevisiae* GRF167 × *S. cerevisiae* S288C. This allows us to understand the impact of varying levels of heterozygosity on adaptation and genome evolution, ranging from none (*S. uvarum* and previous *S. cerevisiae* experiments), to intraspecific heterozygosity (S*. cerevisiae* GRF167 × *S. cerevisiae* S288C), to the most extreme case of interspecific hybrids. *Saccharomycesuvarum* is one of the most distantly related species of *S. cerevisiae* in the *Saccharomyces* clade, separated by 20 My and 20% sequence divergence at coding sites (Kellis et al. 2003; Cliften et al. 2006). Despite this extensive divergence, *S. cerevisiae* and *S. uvarum* are largely syntenic and create hybrids, though less than 1% of spores are viable (Greig 2009). The two species differ in their stress tolerances, for example, *S. cerevisiae* being more thermotolerant, *S. uvarum* being cryotolerant (Almeida et al. 2014). Previous evolution experiments using lab-derived hybrids have revealed novel and/or transgressive phenotypes for ammonium limitation, ethanol tolerance, and growth on xylose (Belloch et al. 2008; Wenger et al. 2010; Piotrowski et al. 2012; Dunn et al. 2013). Notably, Dunn et al. (2013) revealed several LOH events and a repeatable nonreciprocal translocation that produces a gene fusion at the high-affinity ammonium permease *MEP2* after selection in ammonium limitation, offering insight into potential mutational events in the adaptation and/or stabilization of *S. cerevisiae* × *S. uvarum* hybrids.

Here, we evolved these hybrids and diploids in replicate in three nutrient-limited conditions for hundreds of generations. Using whole genome sequencing, we found whole chromosome aneuploidy, genome rearrangements, copy number variants, de novo point mutations, and LOH. We sought to determine how initial heterozygosity affects adaptation to novel conditions and explore whether neutral or selective forces are influencing the resolution of the hybrid genome over time. In particular, we investigated a reoccurring LOH event observed in both intra- and interspecific hybrids and found support for the hypothesis that LOH at this locus is due to selection.

## Results

### Experimental Evolution of Hybrid and Parental Species

An interspecific hybrid was created by crossing *S. cerevisiae* and *S. uvarum* (strains in supplementary table S1, Supplementary Material online) and evolved in continuous culture in the chemostat (Monod 1949; Novick and Szilard 1950a, 1950b). In parallel, homozygous diploid *S. uvarum* and heterozygous diploid *S. cerevisiae* (GRF167 × S288C) were also evolved. Each strain was grown in two or more replicate independent cultures under three different nutrient limitations—glucose, phosphate, and sulfate—for 85–557 generations (median 158) at 30 °C, except for *S. uvarum*, which was unable to achieve steady state in all conditions at 30 °C and so was evolved at 25 °C. The population sizes were largely similar across strains, species, and conditions.

Evolved clones were isolated from each population and subsequently competed individually against the appropriate green fluorescent protein (GFP)-tagged ancestor to gauge relative fitness. As expected, evolved hybrid and parental clones generally exhibit higher fitness than their unevolved ancestor, with typical relative fitness gains between 20% and 30% (tables 1 and 2). To explore whether these fitness gains are general or condition specific, we additionally competed each hybrid clone in the two nutrient-limited conditions in which the clone was not evolved. Results are variable, with some clones having negative or neutral fitness in the alternate conditions, suggesting condition-specific adaptation, and some clones experiencing fitness gains in multiple conditions, suggesting more general growth benefits (table 1). Only one clone exhibited fitness gains in all three nutrient environments, and no clones have a greater fitness gain in an alternate condition than the condition it was evolved in, signifying that clones are largely specifically adapted to the particular condition in which they were evolved.

**Table 1:** Mutations and Fitness of Evolved Hybrid Clones.

Clone	Location	Gene(s)	Mutation	Species	Generations	Relative Fitness ± SE (condition)
Gh1	chrXIII: 852028		Intergenic	cer	125	26.80 ± 0.98 (G); 0.35 ± 1.60 (S); −1.18 (P)
	chrII: 911866..917272	*HXT6/7*	CNV (amplification)	uva		
Gh2	chrIV: 111919	*SNF3*	Nonsynonymous: D114Y	cer	100	28.17 ± 2.18 (G); 10.48 ± 0.78 (S); 11.23 (P)
	chrIII: 51593	*GLK1*	Synonymous: T252T	cer		
	chrIV: 884801..912119	13 genes including *IRC3*	LOH, CNV	uva lost		
	chrII: 912143..917470	*HXT6/7*	CNV (amplification)	uva		
	chrIV	836 genes	CNV (amplification)	cer		
Gh3	chrII: 889421	*IRC3*	nonsynonymous: M333I	uva	124	18.65 ± 0.47 (G); 17.68 ± 3.67 (S); −10.46 (P)
	chrII: 912416..917778	*HXT6/7*	CNV (amplification)	uva		
Ph1	chrV: 269392		Intergenic	cer	103	29.18 ± 1.37 (P); −1.68 ± 0.78 (G); 0.08 ± 0.43 (S)
	chrXIV: 746688		Intergenic	cer		
	chrIV: 1055864	*MHR1*	Nonsynonymous: T218R	cer		
	chrIX	241 genes	LOH, CNV	uva lost, cer amp		
Ph2	chrV: 432778	***GLC7***	Intron	cer	124	25.34 ± 0.24 (P); 15.12 ± 4.66 (G); −2.45 ± 1.16 (S)
	chrVII: 9524	*PDR11*	Nonsynonymous: L383*	uva		
	chrXVI: 232879	MRPL40	Nonsynonymous: V149E	uva		
	chrXIII: 194496	*YML037C*	Nonsynonymous: P306S	uva		
	chrIV: 244399	*YDL114W*	Nonsynonymous: G119C	uva		
	chr IV	836 genes	CNV (amplification)	cer		
Ph3	chrIV: 1055864	*MHR1*	Nonsynonymous: T218R	cer	167	30.03 ± 4.31 (P); 21.39 ± 6.25 (G); NA (S)
	chrIX: 30830..33084	*YIL166C*	CNV (amplification)	cer		
	chrXIII: 0..24562	10 genes including *PHO84*	LOH, CNV	uva lost, cer amp		
	chrIV	836 genes	CNV (amplification)	cer		
Ph4	chrVII: 555885	*RPL26B*	Intron	cer	131	27.02 ± 3.62 (P); 0.68 ± 4.10 (G); 20.46 ± 8.60 (S)
	chrX: 246208	*PHS1*	Nonsynonymous: K206N	cer		
	chrXIII: 324121	*EIS1*	Nonsynonymous: E349*	uva		
	chrIII:0..82687	49 genes	LOH, CNV	cer lost		
	chrXIII:0..221753	112 genes, including *PHO84*	LOH, CNV	uva lost, cer amp		
Ph5	chrXIII: 231731	*PPZ1*	Nonsynonymous: A63S	uva	122	30.24 ± 8.32 (P); −8.20 ± 0.34 (G); 18.20 ± 2.91 (S)
	chrXIII: 0..234112	120 genes, including *PHO84*	LOH, CNV	uva lost, cer amp		
	chrIX:370117..439888	45 genes	LOH, CNV	cer lost		
Ph6	chrVII: 972813	***PFK1***	Nonsynonymous: G308S	cer	111	25.52 ± 3.32 (P); 5.22 ± 2.81 (G); NA (S)
	chrIV	836 genes	CNV (amplification)	cer		
Sh1	chrII:511362..644974; 696397.. 813184	74 genes; 63 genes including *SUL1*	LOH,CNV	cer lost; cer amp	126	33.86 ± 4.60 (S); 3.81 ± 1.17 (G); −15.48 ± 8.55 (P)
	chIV: 680386.. 866667; 866667.. 983774	104 genes; 63 genes	LOH, CNV	uva amp; uva lost		
	chrXVI: 847000.. 948066	49 genes	LOH, CNV	cer lost		
Sh2	chrVII: 936384	*MRPL9*	Nonsynonymous: D167G	cer	268	19.64 ± 4.30 (S); −6.19 ± 1.21 (G); −1.43 ± 6.37 (P)
	chrXVI: 572308	*ICL2*	Nonsynonymous: M247I	uva		
	chrVIII: 116661	***ERG11***	Nonsynonymous: S286C	uva		
	chrII:787389..813,184	11 genes including *SUL1*	CNV (amplification)	cer		
Sh3	chrVI: 162998	*GCN20*	Nonsynonymous: D171Y	cer	132	21.84 ± 1.53 (S); −6.07 ± 1.11 (G); 5.55 ± 4.81 (P)
	chrXIV: 495890	*FKH2*	Synonymous: S418S	uva		
	chrII:786584..813,184	11 genes including *SUL1*	CNV (amplification)	cer		
Sh4	chrXIV: 666675	*ARE2*	Nonsynonymous: I446T	cer	285	27.19 ± 4.33 (S); −6.17 ± 0.51 (G); −20.55 ± 3.30 (P)
	chrXV: 800832	*APC5*	5’-upstream	cer		
	chrIV: 25917	*TRM3*	Synonymous: S201S	cer		
	chrV: 342563		Intergenic	uva		
	chrX: 769768	*SPO77*	Nonsynonymous: D418G	uva		
	chrX: 990873	*LEU3*	5’-upstream	uva		
	chrXII: 192491		Intergenic	uva		
	chrXIV: 25138	*EGT2*	Synonymous: T168T	uva		
	chrII: 770311..813184	22 genes, including *SUL1*	CNV (amplification)	cer		
	chrVIII	321 genes	CNV (amplification)	uva		
Sh5	chrIV: 310881	*RXT3*	Nonsynonymous: P87T	uva	263	46.52 ± 4.94 (S); 6.79 ± 1.35 (G); 3.72 ± 7.23 (P)
	chrVIII: 16911		Intergenic	uva		
	chrII: 786040..813184	11 genes including *SUL1*	CNV (amplification)	cer		
Sh6	chrV: 269392		Intergenic	cer	273	47.52 ± 3.69 (S); 2.60 ± 1.25 (G); 4.47 ± 6.04 (P)
	chrXIV: 746688		Intergenic	cer		
	chrIV: 413046		Intergenic	uva		
	chrII:778942..813,184	14 genes including *SUL1*	CNV (amplification)	cer		
Sh7	chrII: 238875		Intergenic	cer	129	31.44 ± 0.49 (S); −1.87 ± 1.99 (G); 8.74 ± 9.22 (P)
	chrXVI: 490631	*SVL3*	Nonsynonymous: A245V	cer		
	chrXVI: 86106	*YPL245W*	Nonsynonymous: A174D	cer		
	chrII: 273296		Intergenic	uva		
	chrII:737875..813184	42 genes, including *SUL1*	CNV (amplification)	cer		

Point mutations, copy number variants (CNVs), and loss of heterozygosity events (LOH) are recorded for each evolved hybrid clone. Clones are identified by nutrient (G: glucose limitation, P: phosphate limitation, and S: sulfate limitation), an “h” denotes hybrid, and the number indicates its derivation from independent populations. Genes in underline have been found to have point mutations in prior experiments. Note that mutations in the *S. uvarum* genome use *S. uvarum* chromosomes and coordinates. All break points were called by visual inspection of sequencing reads and are thus approximate. Relative fitness is reported with standard error (SE) and the condition the clone was evolved in listed first, followed by the two alternative conditions; several clones are reported without SE due to technical difficulties with replicates.

**Table 2:** Mutations and Fitness of Evolved Parental Clones.

Clone	Location	Gene(s)	Mutation	Species	Generations	Relative Fitness ± SE
Gc1	chrXIV:0..561000; 632250..784333	298 genes; 79 genes	CNV (amplification of chr 14L favoring GRF167; deletion of chr14R)	cer	163	16.42 ± 3.42
	chrV:160000..576874	220 genes	LOH (favors GRF167)			
Gc2	chrV:431750..576874	71 genes	CNV (amplification, favoring GRF167)	cer	167	10.36 ± 0.58
	chrXV:710000..1091291	196 genes	LOH, CNV(monosomy, favoring S288C)			
Gu1	chrXV	597 genes	CNV (whole chromosome amplification)	uva	468	18.03 ± 2.12
	chrII:911925..917281	*HXT6/7*	CNV (amplification)			
	chrXV:385930	*NEL1*	Nonsynonymous: N129I			
	chrII:911909		Intergenic, part of the *HXT6/7* amplification			
Gu2	chrXV	597 genes	CNV (whole chromosome amplification)	uva	486	13.12
	chrII:911925..917281	*HXT6/7*	CNV (amplification)			
	chrIV:100293	*RGT2*	Nonsynonymous: G107V			
	chrV:42093	*FRD1*	Nonsynonymous: G128A			
	chrII:917191	*HXT7*	Synonymous: H53H			
	chrXI:155787		Intergenic			
Pc1	chrXIII:0..39000 (LOH); 0..196628 (CNV: 3 copies); 196628..373000 (CNV: 2 copies)	LOH: 15 genes including *PHO84*; CNV: 201 genes	LOH, CNV (amplification, favoring GRF167)	cer	152	21.22 ± 0.81
Pc2	chrXIII:0..41100 (LOH); 0..196628 (CNV: 3 copies); 196628..373000 (CNV: 2 copies)	LOH: 16 genes including *PHO84*; CNV: 201 genes	LOH, CNV (amplification, favoring GRF167)	cer	149	18.13 ± 1.03
	chrVIII:520349		Intergenic			
Pc3	chrXIII:0..39000 (LOH); 0..196628 (CNV: 3 copies); 196628..373000 (CNV: 2 copies)	LOH: 15 genes including *PHO84*; CNV: 201 genes	LOH, CNV (amplification, favoring GRF167)	cer	127	19.49
Pc4	chrXIII:0..85500 (LOH); 0..196628 (CNV: 3 copies); 196628..373000 (CNV: 2 copies)	LOH: 40 genes including *PHO84*; CNV: 201 genes	LOH, CNV (amplification, favoring GRF167)	cer	132	20.96 ± 1.41
	chrXII: 264000..1078177	437 genes	LOH (favoring S288C)			
	chrXV:1023197	*PIP2*	Nonsynonymous: E6Q			
Pu1				uva	240	−1.68 ± 1.10
Pu2	chrIX:14480	*YPS6*	5’-upstream	uva	234	21.30 ± 0.73
	chrIX: 225314	*SEC6*	Nonsynonymous: I184L			
	chrXIII: 129567	*TCB3*	Nonsynonymous: E625G			
Sc1	chrXIV:0..102000 (CNV: 3 copies); 632000..784333 (CNV: 1 copy); LOH: 100000..784333	48 genes; 79 genes; 367 genes	LOH, CNV (amplification of chr 14L; deletion of chr14R; LOH favoring S288C)	cer	182	38.06 ± 1.75
	chrVIII:207967	*SMF2*	Nonsynonymous: W105S			
	chrXIII:190000..196500	*RRN11*, *CAT2*, *VPS71*	LOH, CNV (deletion, favoring GRF167)			
	chrII:787180..797350	*VBA1*, *SUL1*, *PCA1*	CNV (amplification)			
Sc2	chrXII	578 genes	CNV (whole chromosome amplification, favoring GRF167)	cer	176	40.21 ± 1.33
	chrXII:692000..1078177	193 genes	LOH (favoring GRF167)			
	chrII:773220..813184	18 genes including *SUL1*	CNV (amplification)			
Sc3	chrVI:94104	*FRS2*	Nonsynonymous: V303I	cer	201	41.34 ± 6.77
	chrVIII:308903	*TRA1*	Nonsynonymous: V2048A			
	chrXIV:232266	*POP1*	Nonsynonymous: S477*			
	chrXV:291219	*TLG2*	Nonsynonymous: D286Y			
	chrXV:30986	*HPF1*	Synonymous: T207T			
	chrII:781800..792230	5 genes including *BSD2* and *SUL1*	CNV (amplification)			
Sc4	chrII:275000..813184	289 genes	LOH (favoring GRF167)	cer	190	31.25 ± 6.13
	chrII:788608..795833	*SUL1*, *PCA1*	CNV (amplification)			
	chrXI:517650..666816	68 genes	CNV (amplification)			
	chrXIII:190000..196500	*RRN11*, *CAT2*, *VPS71*	LOH, CNV (deletion, favoring GRF167)			
	chrXIV:632000..784333	79 genes	LOH, CNV (deletion)			
	chrXV: 336700..342000; 342000..1091291	2 genes; 384 genes	LOH (favoring GRF167; favoring S288C)			
	chrIX:23367	*CSS1*	Nonsynonymous: D914N			
Su1	chrX:177350..345680	96 genes including *SUL2*	CNV (amplification)	uva	557	21.8 ± 2.37 (Sanchez, et al. 2017)
	chrXVI:466649	*DIG1*	Nonsynonymous: E49Q			
	chrV:188548		Intergenic			
Su2	chrX:177350..345680	96 genes including *SUL2*	CNV (amplification)			
	chrIV:803704	*KTR3*	5’-upstream			
	chrII:121779	*PIN4*	Nonsynonymous: N263S			
	chrVII:165902	*MPT5*	Nonsynonymous: Q618K			
	chrII:836169	*RSC3*	Synonymous: R4R			
	chrIV:107948	*UFD2*	Synonymous: G691G			
	chrIII:287618		Intergenic			

Point mutations, copy number variants (CNV), and loss of heterozygosity events (LOH) are recorded for each evolved parental clone. Clones are identified by nutrient (G: glucose limitation, P: phosphate limitation, and S: sulfate limitation), by species (“c” denotes *S. cerevisiae*, “u” denotes *S. uvarum*), and the number indicates its derivation from independent populations. Note that mutations in the *S. uvarum* genome use *S. uvarum* chromosomes and coordinates. All break points were called by visual inspection of sequencing reads and are thus approximate. Relative fitness is reported with standard error (SE); several clones are reported without SE due to technical difficulties with replicates.

### Mutations Are Recovered in Both Novel and Previously Observed Gene Targets in Interspecific Hybrids

To identify mutations in the evolved hybrids, we generated whole genome sequencing data for 16 clones from the end points of the evolution experiments (table 1). We thus captured data from a range of nutrient limitations (phosphate: 6; glucose: 3; sulfate: 7) and generations (100–285, median: 154). Each clone had an average of 2.4 point mutations, a number of which have been previously identified in prior *S. cerevisiae* evolution experiments. For example, a nonsynonymous mutation in the *S. cerevisiae* allele of the glucose-sensing gene *SNF3* has been identified in glucose-limited experiments in *S. cerevisiae* (Kvitek and Sherlock 2013; Selmecki et al. 2015). To our knowledge, 20/27 coding point mutations are unique to these experiments (Payen et al. 2016).

In evolved parentals, we again sequenced one clone from the end point of each population. In total, we sequenced 16 clones, 6 from each of the 3 nutrients (2 *S. uvarum* diploids and 4 *S. cerevisiae* diploids), except in glucose limitation in which only 2 *S. cerevisiae* populations were sampled. The generations ranged from 234 to 557 (median: 477) in *S. uvarum*, with an average of 2.83 mutations per clone, and from 127 to 190 (median: 166.5) in *S. cerevisiae*, with an average of 0.9 point mutations per clone (table 2). This discrepancy in point mutations between *S. cerevisiae* and *S. uvarum* may be explained by differences in generation number.

With the limited number of samples we have from hybrid and parental clones, it is difficult to draw any conclusions regarding unique point mutations in hybrids. Furthermore, we have not tested the fitness of these mutations to prove they are adaptive. However, one class of mutations that may be of particular interest in hybrids are genomic mutations that may interact with the mitochondria, as previous work has shown that nuclear–mitochondria interactions can underlie hybrid incompatibility (Lee et al. 2008; Chou and Leu 2010; Meiklejohn et al. 2013). Other studies have found that only the *S. cerevisiae* mitochondria are retained in *S. cerevisiae* × *S. uvarum* hybrids (Antunovics et al. 2005), and we recapitulate these findings, potentially setting the stage for conflicting interactions between the *S. uvarum* nuclear genome and the foreign mitochondria. We observe several mitochondria-related mutations in hybrids in both *S. cerevisiae* and *S. uvarum* alleles. For example, one point mutation, a nonsynonymous mutation in the *S. cerevisiae* allele of the mitochondrial ribosomal protein gene *MHR1*, was seen in two separate clones independently evolved in phosphate limitation. This gene may be of particular interest as it was discovered in a previous screen as being haploproficient (increased fitness of 19%) in hybrids in which the *S. cerevisiae* allele is missing and the *S. uvarum* allele is retained (Lancaster S, Dunham MJ, unpublished data), suggesting that this mutation may alter or disable the *S. cerevisiae* protein in some way. Another example involves the gene *IRC3*, a helicase responsible for the maintenance of the mitochondrial genome, which has a nonsynonymous mutation in the *S. uvarum* allele in clone Gh3 and is deleted in clone Gh2, potentially suggesting that the *S. uvarum* allele is deleterious in the hybrid background. Although our sample size is small, 4/27 point mutations in hybrids are related to mitochondria function compared with 0/26 in parentals and may represent interesting targets for further exploration.

### Copy Number Variants Frequently Involve the Amplification of Nutrient Transporters

**Fig. 1.**
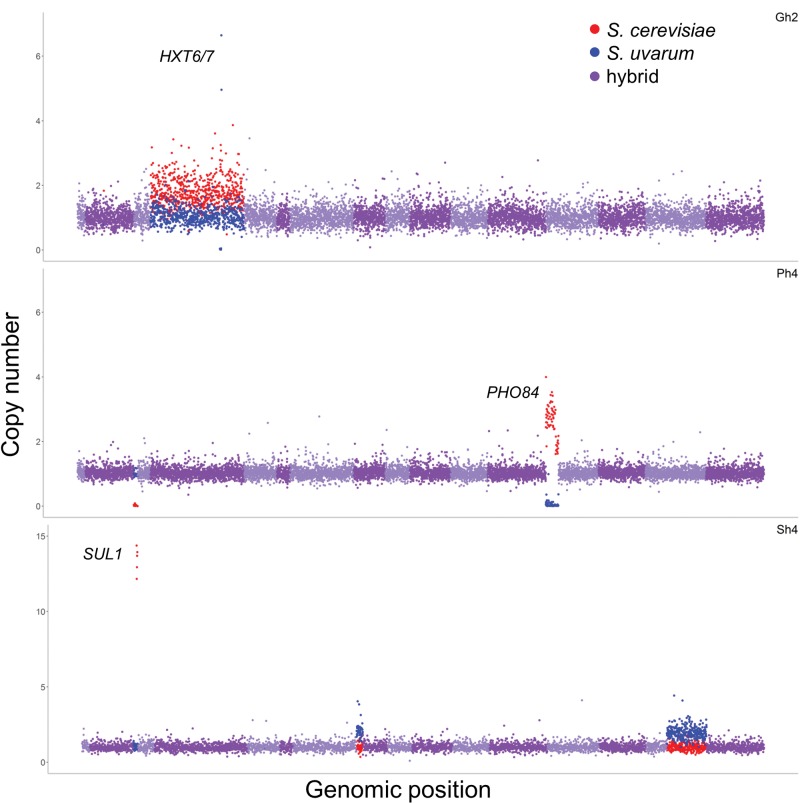
Evolved hybrids exhibit changes in copy number and loss of heterozygosity. Copy number variants are displayed for selected evolved hybrid clones from three nutrient-limited conditions: Gh2, glucose; Ph4, phosphate; and Sh4, sulfate. See additional figures in supplementary fig. S3, Supplementary Material online. Hybrid copy number, determined by normalized sequencing read depth per open reading frame (ORF), is plotted across the genome according to *S. cerevisiae* ORF coordinates to account for three reciprocal translocations between *S. cerevisiae* and *S. uvarum*. Chromosomes are plotted in alternating light and dark purple, red indicates a *S. cerevisiae* copy number variant, and blue indicates a *S. uvarum* copy number variant. Gh2 has a whole chromosome amplification of *S. cerevisiae* chrIV, a small segmental deletion of *S. uvarum* chrIV (non-copy neutral loss of heterozygosity), and an amplification of *S. uvarum HXT6/7*. Ph4 has a small segmental deletion of *S. cerevisiae* chrIII (non-copy neutral loss of heterozygosity) and an amplification of *S. cerevisiae* chrXIII with corresponding deletion of *S. uvarum* chrXIII (copy neutral loss of heterozygosity). Sh4 has an amplification of *S. cerevisiae SUL1* and a whole chromosome amplification of *S. uvarum* chrVIII (note, there is a reciprocal translocation between chrVIII and chrXV in *S. uvarum* relative to *S. cerevisiae*). Note that Sh4 is plotted on a different scale. For specific coordinates of copy number variants, see table 1.

Yeasts in both natural and artificial environments are known to frequently experience changes in copy number, ranging from single genes to whole chromosomes (Dunham et al. 2002; Gresham et al. 2008; Dunn et al. 2012; Kvitek and Sherlock 2013; Payen et al. 2014; Selmecki et al. 2015; Sunshine et al. 2015; Zhu et al. 2016). This holds true in our evolution experiments: We observe copy number changes across all genetic backgrounds (fig. 1; supplementary figs. S1–S3, Supplementary Material online). Clones were compared with array comparative genomic hybridization of populations to confirm that clones are representative of populations (see Materials and Methods). The evolved hybrid clones displayed an average of 1.5 copy number variants (CNVs) per clone (fig. 1, table 1; Supplementary fig. S3, Supplementary Material online), as defined by the number of segmental or whole chromosome amplifications/deletions (though it is likely that some of these CNVs were created in the same mutational event). The evolved *S. cerevisiae* clones had an average of 1.5 CNV per clone and the evolved *S. uvarum* clones had an average of 1 CNV per clone (table 2; supplementary figs. S1 and S2, Supplementary Material online). The most common event across nutrient limitations in the interspecific hybrids was an amplification of the *S. cerevisiae* copy of chromosome IV, which occurred in four independent hybrid clones (three in phosphate limitation, one in glucose limitation; supplementary fig. S3, Supplementary Material online). Several other characteristic rearrangements occurred in the evolved *S. cerevisiae* clones, including the amplification of the left arm of chromosome 14 accompanied by segmental monosomy of the right arm of chromosome 14, an event seen previously in other evolved populations (Dunham et al. 2002; Gresham et al. 2008; Sunshine et al. 2015). All copy number events in *S. cerevisiae* had break points at repetitive elements known as Ty elements, except those located on chrII, which may be mediated by another mechanism (Brewer et al. 2015). In contrast, copy number variants in the hybrid were rarely facilitated by repetitive elements, perhaps in part because *S. uvarum* has no full length Ty elements; however, why *S. cerevisiae* Ty elements and long terminal repeat (LTR) sequences from either genome were not utilized remains unknown.

**Fig. 2.**
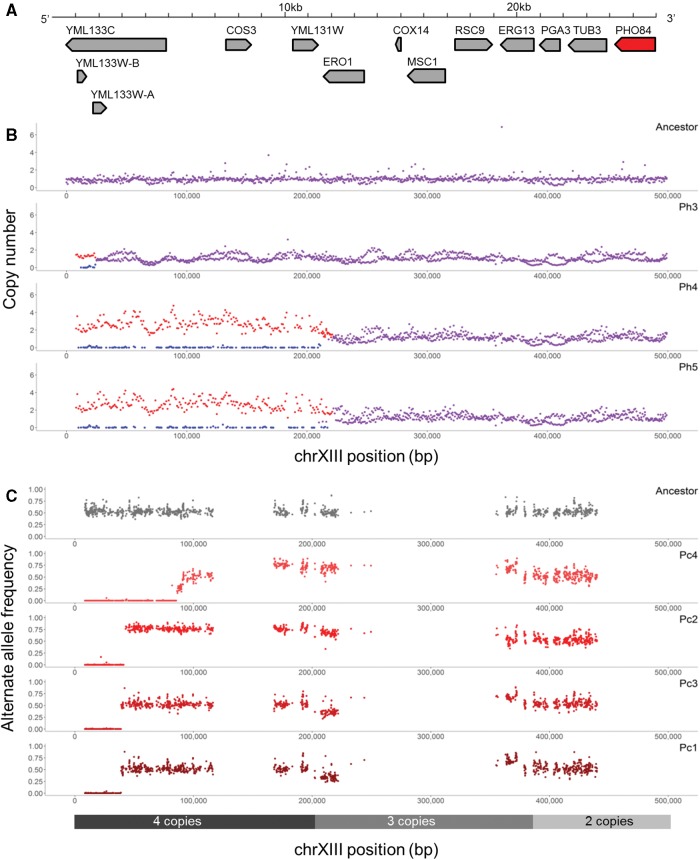
Repeated loss of heterozygosity at the *PHO84* locus in intra- and interspecific hybrids. (*A*) The 25-kb region extending from the left telomere of chromosome XIII to the high-affinity phosphate transporter gene *PHO84*. (*B*) Copy number is plotted across part of chromosome XIII in the hybrid ancestor and three evolved hybrid clones in phosphate limitation (clone indicated in upper right corner). Red shows the *S. cerevisiae* allele, blue shows the *S. uvarum* allele, and purple shows where both species exhibit the same copy number. Note: 8 kb of telomere sequence is removed due to repetitive sequence. (*C*) Alternate allele frequency is plotted for a portion of chromosome XIII in the ancestor and four evolved *S. cerevisiae* clones in phosphate limitation (clone indicated in upper right corner). All evolved *S. cerevisiae* clones exhibit a loss of heterozygosity at the telomeric portion of chromosome XIII (loss of S288C, amplification of GRF167), as illustrated by an allele frequency of zero compared with the ancestor. Regions of heterozygosity are interspersed with regions of homozygosity, as one of the parents of the diploid was itself the product of a cross between strains FL100 and S288C, and the other parent was S288C. Regions of heterozygosity are due to FL100 haplotypes. *S. cerevisiae* copy number for the four evolved clones is shown below; the ancestor is diploid across the chromosome (also see table 2, supplementary fig. S1, Supplementary Material online).

Frequently in nutrient-limited evolution experiments, copy number variants involve amplification of the nutrient-specific transporter, and indeed, we also observed amplification of these transporters in many of the clones. In sulfate limitation, the *S. cerevisiae* allele of the high-affinity sulfate transporter gene *SUL1* is amplified in 7/7 hybrid clones and 4/4 *S. cerevisiae* clones (fig. 1, tables 1 and 2; supplementary figs. S1 and S3, Supplementary Material online). Interestingly, *SUL2* is the preferred sulfate transporter in *S. uvarum* (Sanchez et al. 2017) and was not observed to be amplified in the evolved hybrids (supplementary fig. S2 and table S2, Supplementary Material online). In glucose limitation, previous *S. cerevisiae* evolution experiments found frequent amplification of the high-affinity glucose transporter genes *HXT6/7* (Brown et al. 1998; Dunham et al. 2002; Gresham et al. 2008; Kao and Sherlock 2008; Kvitek and Sherlock 2011). In our experiments, the *S. uvarum* alleles of the *HXT6/7* transporters are amplified in 3/3 hybrid clones and both *S. uvarum* clones but are not amplified in evolved *S. cerevisiae* clones. This is suggestive that the *S. uvarum HXT6/7* alleles confer a greater fitness advantage compared with *S. cerevisiae*; alternatively, the genomic context could be more permissive to amplification in *S. uvarum* (fig. 1, tables 1 and 2; supplementary figs. S1–S3, Supplementary Material online). Finally, in phosphate limitation, the *S. cerevisiae* copy of the high-affinity phosphate transporter gene *PHO84* is amplified, and the *S. uvarum* allele is lost in 3/6 hybrid clones in an event known as LOH (figs. 1 and2, table 1; supplementary fig. 3, Supplementary Material online). Intriguingly, the evolved *S. cerevisiae* clones also display LOH and accompanied amplification favoring the allele derived from strain GRF167 over the S288C allele in 4/4 clones (fig. 2, table 2). All hybrid clones carry the “preferred” GRF167 *S. cerevisiae* allele, as this was the allele used to create the de novo hybrid.

### Loss of Heterozygosity Is a Common Event in Heterozygous Evolving Populations

Selection on heterozygosity, as a LOH event could represent, is an underappreciated source of adaptation in microbial experimental evolution, as typical experiments evolve a haploid or homozygous diploid strain asexually and, as a result, have little variation to select upon. LOH is observed in natural and industrial hybrids (Albertin and Marullo 2012; Louis et al. 2012; Pryszcz et al. 2014; Wolfe 2015; Schroder et al. 2016), but here we document its occurrence in both intra- and interspecific hybrids in the laboratory as a result of short-term evolution (also see (Dunn et al. 2013; Burke et al. 2014)). LOH is observed across all nutrient conditions, with 13 independent LOH events detected in *S. cerevisiae* and 9 independent events documented in the hybrids (figs. 1 and2, tables 1 and 2; supplementary figs. S1 and S3, Supplementary Material online). It thus appears that this type of mutational event is both common and can occur over short evolutionary timescales.

The LOH event can result in copy-neutral (where one allele is lost and the other allele is amplified) or non-neutral chromosomal segments (where one allele is lost, rendering the strain hemizygous at that locus) and can favor the retention of either allele. In *S. cerevisiae*, there is a bias in resolution where LOH events favor retaining the GRF167 allele over the S288C allele (10/13 events, *P* = 0.0169; table 2; supplementary fig. S4, Supplementary Material online). One unique case in clone Sc4 has a small approximately 5 kb LOH event on chrXV favoring GRF167, which switches to favoring S288C for the rest of the chromosome. The retention of *S. cerevisiae* is slightly more common in the hybrids (5/9 events, *P* = 1.0; table 1; supplementary fig. S3, Supplementary Material online), though not as drastic as the observed genome resolution in the hybrid *S. pastorianus*, where LOH favors *S. cerevisiae* over *S. eubayanus* (Nakao et al. 2009). The size of the event ranges from approximately 25 kb to the whole chromosome level in the hybrids and from 5 kb to 540 kb in *S. cerevisiae*. Where LOH is accompanied by an amplification event, the LOH event always occurs first and does not share break points with the amplification event. Unlike many CNV events, most LOH events do not appear to be mediated by existing repetitive sequence such as a transposable element in the hybrid or *S. cerevisiae* and are most likely a product of break-induced replication or mitotic gene conversion, with break-induced replication as the favored method as all events extend to the telomere (Hoang et al. 2010). The exceptions are in hybrid clones Ph4, Ph5, and Sh1, where there is a non-copy neutral loss of *S. cerevisiae* mediated by a Ty element or LTR, and *S. cerevisiae* clones Sc1 and Sc4, where there is a 6.5-kb deletion of the S288C allele flanked by two Ty elements.

LOH events in hybrids could signify several ongoing processes in hybrid genome evolution: LOH regions may represent 1) loci with incompatibilities, 2) selection on existing variation, or 3) genetic drift eroding genomic segments. It is impossible to definitively rule out any of these hypotheses without further experimentation; however, there are several arguments disfavoring the incompatibility hypothesis. First, although our sample size is modest, failing to see repeated LOH events across nutrient conditions may indicate a lack of general incompatibility between species (although we cannot preclude condition-specific incompatibility, Hou et al. 2015; Piatkowska et al. 2013). This is consistent with previous studies in yeast, which suggest that classic Dobzhanksy–Muller incompatibilities are rare (Liti et al. 2006; Greig 2009; Hou et al. 2014). Furthermore, LOH events observed in evolved *S. cerevisiae* suggest that this mutation type is not unique to interspecific hybrids. Instead, repeated events within a particular condition, such as the repeated LOH at *PHO84* in phosphate limitation or the 6.5 kb segment on chrXIII in sulfate limitation, suggest that these events are beneficial and are indeed selection on one allele over the other.

### LOH Is Driven by Selection on One Allele

To test the hypothesis that LOH events provide a selective advantage, we used allele replacement, in which the allele of one species/strain is replaced with the allele of the other species/strain in an otherwise isogenic background. We tested this hypothesis using the most commonly seen LOH event, LOH at *PHO84*. Although the region extends from 25 kb to 234 kb in length in the hybrids and from 40 kb to 85 kb in *S. cerevisiae* and thus includes many genes, *PHO84* was a prime candidate driving this event. *PHO84* is one of only ten genes encompassed in the region extending from the telomere to the break point of the shortest LOH event and is included in every other LOH event on chromosome XIII (fig. 2). It is a high-affinity phosphate transporter responsible for inorganic phosphate uptake in high and low phosphate conditions (Wykoff and O'Shea 2001), and previous work identified a point mutation in *PHO84* (an alanine to valine substitution at the 5ʹ end of the gene), which increased fitness by 23% in phosphate-limited conditions (Sunshine et al. 2015). Finally, prior work with other nutrient transporters has shown amplification of nutrient transporters to be a key event in adapting to nutrient-limited conditions.

We thus selected a region of approximately 2.5 kb encompassing the *PHO84* ORF, its promoter, and 3ʹ-UTR (Nagalakshmi et al. 2008; Yassour et al. 2009; Cherry et al. 2012). We created allele replacement strains using the two alleles of *S. cerevisiae* in a *S. cerevisiae* diploid background; the two alleles are 99.1% identical in this region (supplementary fig. S5, Supplementary Material online) and each strain is identical to the ancestral strain used in our evolution experiments except at the *PHO84* locus. The *S. cerevisiae* ancestor carries one copy of GRF167 (preferred) and one copy of S288C (“un-preferred”), so named due to which allele was retained and amplified in the evolved clones. To measure any resultant changes in fitness, we competed each strain individually against a fluorescent ancestral strain and measured which strain overtook the culture. Relative fitness is thus defined as the growth advantage per generation. Two copies of the un-preferred allele decreased relative fitness by −5.31% (±1.86), whereas two copies of the preferred allele increased relative fitness by 9.93% (±0.27). This displays an overall difference in relative fitness of 15.24% between the un-preferred and preferred alleles. By comparing the fitness of these allele replacement strains with the evolved clones (table 2), the allele replacement does not fully recapitulate the fitness gain observed in the evolved clone. One explanation is that the additional mutations present in the evolved strains also contribute to their total fitness. Another explanation could be the increased copy number of the *PHO84* region that we see in these evolved clones. To further explore this fitness difference, we cloned the GRF167 allele onto a low copy number plasmid and transformed the allele replacement strain carrying two preferred *S. cerevisiae* alleles to simulate increased copy number of *PHO84* and saw only a minimal fitness increase of 1.76% (±1.06; note all fitness competitions involving plasmids were done in conjunction with empty plasmids to take into account any fitness effects from the plasmid itself). This supports the conclusion that relative fitness gains in the evolved clone are largely due to the loss of the S288C allele, and selection and amplification of the GRF167 allele, with little additional benefit from further amplification. It could also be the case that co-amplification of other genes in the segment is required to attain the full benefit, as previously observed by the contribution of *BSD2* to the *SUL1* amplicon in sulfate limitation (Sunshine et al. 2015; Payen et al. 2016).

To understand the fitness effects of LOH and amplification in the hybrids, we generated hybrid strains with varying numbers of *S. cerevisiae* GRF167 *PHO84* alleles. Unfortunately, we were unable to obtain a successful strain carrying the preferred *S. cerevisiae* allele in a *S. uvarum* background; however, we were able to generate a *S. uvarum PHO84* knockout strain, thus creating a hybrid with one copy of *S. cerevisiae PHO84*. When combined with a low copy plasmid carrying the same allele, this strain effectively has two or more copies of *S. cerevisiae PHO84* in a hybrid background. This hybrid strain can serve as a proxy for the LOH event observed in the evolution experiments and has a relative fitness gain of 25.57% (±2.88). The ancestral hybrid with the same plasmid (effectively 1 *S. uvarum* allele and 2 or more *S. cerevisiae* alleles) has a relative fitness gain of 12.53% (±1.31). The difference between these two hybrids suggests that while amplification is beneficial, the highest fitness is achieved with the loss of the *S. uvarum* allele (*P* = 0.0061).

Together, these results support the conclusion that the *S. cerevisiae* GRF167 allele is preferred over the S288C allele and that *S. cerevisiae* alleles are preferred over the *S. uvarum* allele in the hybrid and, hence, that the LOH events seen in both intra- and interspecific hybrids are the product of selection.

## Discussion

In summary, we sought to understand forces underlying genome stabilization and evolution in interspecific and intraspecific hybrids as they adapt to novel environments. We evolved and sequenced clones from 16 hybrid populations and 16 parental populations to reveal a variety of mutational events conferring adaptation to 3 nutrient-limited conditions. Of particular note, we find LOH in both evolved intraspecific and interspecific hybrid clones in all nutrient environments, potentially signifying areas where selection has acted on preexisting variation present in the ancestral clone. We used an allele replacement strategy to test this hypothesis for a commonly repeated LOH event and show that selection is indeed driving the homogenization of the genome at this locus. Although other studies in natural, industrial, and lab-evolved isolates have observed LOH, we present the first empirical test of the causal evolutionary forces influencing these events. This work can begin to help us understand past hybridization events and subsequent genome resolution in hybrids in natural and artificial systems.

### Similarities and Differences between Intra- and Interspecific Hybrids

Although our sample size is modest, we observe several interesting trends when comparing *S. uvarum* clones (homozygous), *S. cerevisiae* clones (intraspecific hybrid and previously published homozygous), and interspecific hybrid clones. First, theory and previous research predict the interspecific hybrid may experience more genome instability in the form of chromosomal rearrangements and CNV events (Xiong et al. 2011; Lloyd et al. 2014; Chester et al. 2015; Mason and Batley 2015). Instead, in our work, the interspecific hybrid and *S. cerevisiae* clones experience the same number of CNV events (both 1.5 CNVs/clone). However, the mechanism of CNV formation seems to differ between the hybrid and *S. cerevisiae* clones. Whereas *S. cerevisiae* CNV break points typically occur at transposable elements in our study, and previous studies (Dunham et al. 2002; Gresham et al. 2008; Fedoroff 2012), the interspecific hybrid CNVs do not utilize transposable elements, although they obviously share the same sequence background as the *S. cerevisiae* clones (albeit in one copy). Whether this difference is due to the absence of full-length transposable elements in *S. uvarum* is unknown, but this could potentially explain the lower number of CNV events in *S. uvarum* clones (1 CNV/clone) and presents an intriguing direction for future study.

### The Predictability of Evolution

We now have many examples of predictable evolution in natural systems (Losos et al. 1998; Rundle et al. 2000; Elmer and Meyer 2011; Conte et al. 2012; Jones et al. 2012; Martin and Orgogozo 2013; Wessinger and Rausher 2014), and in laboratory experimental evolution, in which there often appears to be a limited number of high fitness pathways that strains follow when adapting to a particular condition (Ferea et al. 1999; Woods et al. 2006; Gresham et al. 2008; Burke et al. 2010; Salverda et al. 2011; Kawecki et al. 2012; Kvitek and Sherlock 2013; Lang and Desai 2014). For example, it is well established that amplifications of nutrient transporters are drivers of adaptation in evolution in nutrient-limited conditions. Previous work in our group has particularly focused on the amplification of the high-affinity sulfate transporter gene *SUL1* in sulfate-limited conditions, which occurs in almost every sulfate-limited evolution experiment and confers a fitness advantage of as much as 40% compared with the unevolved ancestor strain. The amplification of phosphate transporters has been markedly less common, and thus drivers of adaptation in this condition have been less clear. Gresham et al. (2008) identified a whole chromosome amplification of chrXIII in one population. In a follow-up study, Sunshine et al. (2015) found whole or partial amplification of chrXIII in 3/8 populations. A genome-wide screen for segmental amplifications found a slight increase in fitness for a small telomeric segment of chromosome XIII, and a A49V point mutation in *PHO84* was observed to increase fitness by 23%. However, screens by Payen *et al.* (2016) showed that although *PHO84* is recurrently mutated in various experiments, it showed no benefit when amplified or deleted in phosphate-limited conditions. Finally, additional evolution experiments recapitulated the point mutation seen in Sunshine et al. (2015) in 24/32 populations and saw amplification of *PHO84* in 8/32 populations (Miller A, Dunham MJ, unpublished data). It is important to note that all these experiments used a strain background derived from S288C or CEN.PK, both of which carry the same (un-preferred) *PHO84* allele.

In our work, we observed amplification of the *S. cerevisiae* GRF167 allele of *PHO84* in 4/4 *S. cerevisiae* clones from 4 populations and 3/6 hybrid clones from 6 populations. This amplification was always preceded by the loss of the S288C allele in *S. cerevisiae* clones, and the LOH break points are never shared with the amplification break points. There is a 15% fitness difference between carrying two copies of the S288C allele of *PHO84* compared with carrying two copies of the GRF167 allele of *PHO84*, and additional copies of the GRF167 allele do not provide substantial further fitness gains. The two alleles differ by several noncoding changes, and three nonsynonymous substitutions: a mutation from glutamic acid to aspartic acid (E229D), leucine to proline (L259P), and leucine to glutamine (L556Q), the latter two of which are considered “nonconservative” protein mutations due to changes in hydrophobicity and structure (supplementary fig. S5, Supplementary Material online). Intriguingly, the L259P mutation has actually been previously identified as being responsible for resistance to tetrachloroisophthalonitrile and partial resistance to pentachlorophenol in a QTL study of small-molecule drugs (Perlstein et al. 2007). Indeed, Perlstein *et al.* found proline at residue 259 to be conserved across fungal species, and even in orthologous human xenobiotic transporters, likely because proline-induced kinks in transmembrane spans have been shown to be essential to protein function (Cordes et al. 2002; Perlstein et al. 2007). It thus appears that amplification of *PHO84* has been less predictable, as the S288C allele does not confer a fitness advantage unless mutated, per Sunshine et al. (2015). Together, these results imply that strain background can constrain adaptive pathways.

In hybrids, the amplification of the *S. cerevisiae* segment occurred in conjunction with the loss of the *S. uvarum* allele. Hybrid strains with the LOH had a 25.57% relative fitness gain, whereas hybrid strains with amplification of the *S. cerevisiae PHO84* allele without the LOH had a 12.53% fitness gain. Thus, LOH confers an additional 13.04% fitness gain, showing that selection for LOH has a larger impact on fitness than amplification alone. Note that *S. uvarum* does have proline at residue 259, like the preferred GRF167 allele, and differs from both *S. cerevisiae* alleles at the other two coding substitutions (229N and 556K), but the amino acid and noncoding divergence is too high to speculate what substitutions are responsible for the selection of the GRF167 allele (supplementary fig. S6, Supplementary Material online). Why the loss of one allele is more beneficial remains unclear, as *PHO84* is thought to function as a monomer (Bun-Ya et al. 1991), but it may be due to competition for cell wall space or negative interactions with other genes in the *PHO* pathway (Mouillon and Persson 2006).

The infusion of variation created by hybridization provides new templates for selection to act upon, which can be more important than either point mutations or copy number variants alone. Our work shows that outcrossing need not be common to have long-lasting effects on adaptation. This implication is particularly relevant in yeast, where outcrossing may occur quite rarely followed by thousands of asexual generations (Ruderfer et al. 2006; Greig and Leu 2009; Liti 2015).

### Applications to Other Hybrids and Cancer

The observation that LOH occurs in hybrid genomes is increasingly documented (Louis et al. 2012; Borneman et al. 2014; Soltis et al. 2014; Marcet-Houben and Gabaldon 2015; Pryszcz et al. 2014; Schroder et al. 2016), although the reason(s) for this type of mutation has been unresolved. As most examples stem from allopolyploid events that occurred millions of years ago, understanding why LOH is important in hybrid genome evolution is difficult. Cancer cells are also known to experience LOH, sometimes involved in the inactivation of tumor suppressor genes, leaving only one copy of the gene that may be mutated or silenced (Thiagalingam et al. 2001; Tuna et al. 2009; Lapunzina and Monk 2011). Data support the conclusion that LOH events are selected for during tumor development, as many LOH events involve specific chromosomal segments (Thiagalingam et al. 2001), although the underlying molecular and genetic reasons for selection is an open debate (Ryland et al. 2015).

Here, we experimentally demonstrate that LOH can occur in homoploid interspecific hybrids as well as in intraspecific hybrids. These events occur within a few hundred generations and are common mutations, more common on average in the intraspecific hybrid (1.3 events/clone) than the interspecific hybrid (0.56 events/clone). Interestingly, the LOH events do not share break points with the CNV events in *S. cerevisiae*; instead, they appear to occur independently and to precede any subsequent amplification (amplification occurs following 9/13 LOH events). Competition assays with the *PHO84* locus provide support that LOH itself may be more beneficial than amplification or at least increase the selective benefit of amplification events. The observation that LOH in intraspecific hybrids occurs independently from copy number change provides different opportunities for adaptation to novel conditions.

Other cases of LOH, like the copy number neutral events observed in Ph4, Ph5, and Sh4, all of which favor the retention of the *S. uvarum* allele (supplementary fig. S3, Supplementary Material online), may be due to hybrid incompatibility within a particular protein complex, other epistatic interactions (Piatkowska et al. 2013), or neutral processes. We furthermore discover examples where one species allele appears to be preferred over the other without LOH, such as the repeated amplification of the *S. uvarum* high-affinity glucose transporters *HXT6/7*. When one species allele is amplified and the other is not amplified, one explanation is that the local sequence context can permit or deny amplification. In the case of *HXT6/7*, previous experiments in *S. cerevisiae* have shown that amplification of *HXT6/7* is quite common (Brown et al. 1998; Dunham et al. 2002; Gresham et al. 2008; Kao and Sherlock 2008; Kvitek and Sherlock 2011), thus suggesting that when given a choice between this locus in *S. cerevisiae* or *S. uvarum* in the hybrid, the preferred allele is indeed *S. uvarum*, though more subtle differences in rate cannot yet be ruled out. A similar scenario is observed with *S. cerevisiae SUL1* (Sanchez et al. 2017). Together, our results show that the heterozygosity supplied by hybridization is an important contributor to adaptive routes explored by populations as they adapt to novel conditions.

Although we cannot generalize our results from the *PHO84* locus across the many other LOH events discovered in our hybrids and *S. cerevisiae*, in the future, we can use similar methodology to explore whether positive selection always drives LOH or whether other explanations such as incompatibility resolution contribute as well. Future experiments might also utilize a high throughput method to explore segmental LOH in hybrids at a genome-wide scale, similar to ongoing experiments at the gene level (Lancaster S, Dunham MJ, unpublished data). Although our sample size is moderate, this is a novel and necessary step in understanding forces underlying hybrid genome stabilization and highlighting an underappreciated mechanism of hybrid adaptation.

### Conclusions

The mutation events we observe in our experimentally evolved hybrids are in many ways quite representative of mutations observed in ancient hybrid genomes, suggesting that hybrid genome stabilization and adaptation can occur quite rapidly (within several hundred generations). Furthermore, our results illustrate that the infusion of variation introduced by hybridization at both the intra- and inter-species level can increase fitness by providing choices of alleles for selection to act upon, even when sexual reproduction is rare. This may be particularly important for leveraging existing variation for agricultural and industrial processes and as climate change potentially increases natural hybridization (Kelly et al. 2010; Hoffmann and Sgro 2011; Muhlfeld et al. 2014).

## Materials and Methods

### Strains

A list of strains used in this study is included in supplementary table S1, Supplementary Material online. All interspecific hybrids were created by crossing a *ura3 LYS2* haploid parent to a *URA3 lys2* haploid parent of the other mating type, plating on media lacking both uracil and lysine, and selecting for prototrophs. The *S. cerevisiae* strain background, known as “GRF167”, is itself a cross between FL100 and the genomic type strain S288C (data not shown). GRF167 was chosen as a strain background for simultaneous work investigating transposable elements during experimental evolution, which will be addressed in a future study.

### Evolution Experiments

Continuous cultures were established using media and conditions previously described (Gresham et al. 2008; Sanchez et al. 2017). Detailed protocols and media recipes are available at http://dunham.gs.washington.edu/protocols.shtml (last accessed March 6, 2017). Samples were taken daily and measured for optical density at 600 nm and cell count; microscopy was performed to check for contamination; and archival glycerol stocks were made daily. An experiment was terminated when contamination, growth in tubing, or clumping appeared (number of generations at the end point for each population are presented in tables 1 and 2). Samples from each end point population were colony purified to yield two clones for further study.

### Array Comparative Genomic Hybridization

Populations from the end point of each evolution were analyzed for copy number changes using array comparative genomic hybridization following the protocol used in Sanchez et al. (2017).

### Sequencing

DNA was extracted from overnight cultures using the Hoffman–Winston protocol (Hoffman and Winston 1987) and cleaned using the Clean & Concentrator kit (Zymo Research). Nextera libraries were prepared following the Nextera library kit protocol and sequenced using paired end 150 bp reads on the Illumina NextSeq 500 machine (sequencing coverage in supplementary table S2, Supplementary Material online). The reference genomes used were *S. cerevisiae* v3 (Engel et al. 2014), *S. uvarum* (Scannell et al. 2011), and a hybrid reference genome created by concatenating the two genomes. Sequence was aligned to the appropriate reference genome using bwa v0.6.2 (Li and Durbin 2009) and mutations were called using GATK (McKenna et al. 2010) and samtools 0.1.19 (Li et al. 2009). Mutations in evolved clones were filtered in comparison with the ancestor to obtain de novo mutations. All mutations were first visually inspected using Integrative Genomics Viewer (Robinson et al. 2011). Subsequently, point mutations in the hybrids were confirmed with Sanger sequencing (supplementary table S3, Supplementary Material online). Copy number variants were visualized using DNAcopy for *S. cerevisiae* and *S. uvarum* (Seshan and Olshen 2016). LOH events were called based on sequencing coverage in the hybrids and by identifying homozygous variant calls in *S. cerevisiae*. All break points were called by visual inspection of sequencing reads and are thus approximate.

### Fitness Assays

The pairwise competition experiments were performed in 20 ml chemostats (Miller and Dunham 2013). Each competitor strain was cultured individually until steady state was reached and then was mixed 50:50 with a GFP-tagged ancestor. Each competition was conducted in at least two biological replicates for approximately 15 generations after mixing. Samples were collected and analyzed twice daily. The proportion of GFP+ cells in the population was detected using a BD Accuri C6 flow cytometer (BD Biosciences). The data were plotted with ln [dark cells/GFP+ cells] versus generations. The relative fitness coefficient was determined from the slope of the linear region.

### Strain Construction

Allele replacements for the *PHO84* locus were done following the protocol of the Caudy lab with further modifications described here. The native locus was replaced with *Kluyveromyces lactis URA3*. The *pho84Δ*::*URA3* strain was grown overnight in 5 ml of C-URA media, then inoculated in a flask of 100 ml yeast extract peptone dextrose (YPD) and grown to an optical density of 0.6–0.8. Cells were washed then aliquoted. 275 µl of transformation mix (35 µl 1 M lithium acetate, 240 µl of 50% 3500 polyethylene glycol), 10 µl of salmon sperm, and approximately 3 µg of polymerase chain reaction product were added to the cell pellet. It was incubated at 37 °C (*S. uvarum*) or 42 °C (*S. cerevisiae*) for 45 min, then plated to YPD. It was replica plated to 5-fluoroorotic acid the following day, and colonies were tested for the gain of the appropriate species allele. The GRF167 allele was cloned into the pIL37 plasmid using Gibson assembly (Gibson et al. 2009). Correct assembly was verified by Sanger sequencing. All primers used can be found in supplementary table S3, Supplementary Material online.

## Supplementary Material

Supplementary data are available at *Molecular Biology and Evolution* online.

## Supplementary Material

Supplementary Data
