# Supplementary material for: Loss of Heterozygosity Drives Adaptation in Hybrid Yeast
Source: Mol Biol Evol. 2017 Mar 29;34(7):1596–612. doi: 10.1093/molbev/msx098 (PMC5455960; doi:10.1093/molbev/msx098)
Supplement: Supplementary Data [file msx098_Supp.pdf]

Supplemental Table 1: Strain list

Strain	Identifier (see Tables 1, 2)	Genotype	Species	Notes	Strain background
YMD139		*lys2Δ0/+ +/ura3-167 MATa/MATα*	cer	Ancestor of *S. cerevisiae* evolved diploids (high Ty)	GRF167 x S288C
YMD140		*lys2Δ0/+ +/ura3-167 MATa/MATα*	cer	Ancestor of *S. cerevisiae* evolved diploids	GRF167 x S288C
YMD129		*HOΔ*::kanMX/*HOΔ*::kanMX *lys2-1/+ +/ura3-167 MATa/MATα*	hybrid	Ancestor of evolved hybrids	GRF167 x CBS7001
YMD130		*HOΔ*::kanMX/*HOΔ*::kanMX *lys2-1/+ +/ura3-167* (high Ty) *MATa/MATα*	hybrid	Ancestor of evolved hybrids (high Ty)	GRF167 x CBS7001
YMD366		*HOΔ*::kanMX /*HOΔ*::kanMX *lys2-1/+ ura3Δ*::cloNAT/+ *MATa/MATα*	uva	Ancestor of *S. uvarum* evolved diploids	CBS7001
YMD35	Pu1		uva	Phosphate limitation	YMD366
YMD92	Pu2		uva	Phosphate limitation	YMD366
YMD18	Pc3		cer	Phosphate limitation	YMD140
YMD77	Pc1		cer	Phosphate limitation	YMD140
YMD66	Pc4		cer	Phosphate limitation	YMD139
YMD98	Pc2		cer	Phosphate limitation	YMD139
YMD30	Ph1		hybrid	Phosphate limitation	YMD130
YMD39	Ph2		hybrid	Phosphate limitation	YMD130
YMD90	Ph3		hybrid	Phosphate limitation	YMD129
YMD101	Ph5		hybrid	Phosphate limitation	YMD130
YMD741	Ph4		hybrid	Phosphate limitation	YMD129
YMD744	Ph6		hybrid	Phosphate limitation	YMD129
YMD723	Gu1		uva	Glucose limitation	YMD366
YMD725	Gu2		uva	Glucose limitation	YMD366
YMD34	Gc2		cer	Glucose limitation	YMD140
YMD23	Gc1		cer	Glucose limitation	YMD139
YMD726	Gh1		hybrid	Glucose limitation	YMD129
YMD737	Gh2		hybrid	Glucose limitation	YMD130
YMD739	Gh3		hybrid	Glucose limitation	YMD130
YMD720	Su1		uva	Sulfate limitation	YMD366
YMD721	Su2		uva	Sulfate limitation	YMD366
YMD31	Sc3		cer	Sulfate limitation	YMD140
YMD83	Sc2		cer	Sulfate limitation	YMD140
YMD105	Sc1		cer	Sulfate limitation	YMD139
YMD21	Sc4		cer	Sulfate limitation	YMD139
YMD751	Sh2		hybrid	Sulfate limitation	YMD130
YMD752	Sh3		hybrid	Sulfate limitation	YMD130
YMD754	Sh4		hybrid	Sulfate limitation	YMD130
YMD747	Sh1		hybrid	Sulfate limitation	YMD129
YMD749	Sh7		hybrid	Sulfate limitation	YMD129
YMD858	Sh5		hybrid	Sulfate limitation	YMD129
YMD861	Sh6		hybrid	Sulfate limitation	YMD129
YMD3348		*ura3-167/+ MATa/MATα*	cer	2 preferred *S. cerevisiae* alleles at *PHO84*	
YMD3349		*PHO84Δ*::Sc*PHO84*(un-preferred allele)/*PHO84Δ*::Sc*PHO84*(un-preferred allele) *ura3-167/+ MATa/MATα*	cer	2 un-preferred *S. cerevisiae* alleles at *PHO84*	
YMD3350		*PHO84Δ*::Sc*PHO84*(un-preferred	cer	1 preferred, 1 un-	

		allele)/+ *ura3-167*/+ *MATa/MATα*		preferred *S. cerevisiae* allele at *PHO84*	
YMD3351		*ura3-167/ura3-167 MATa/MATα +pIL37-PHO84-URA3*	cer	3+ preferred *S. cerevisiae* alleles at *PHO84*	
YMD3352		*ura3-167/ura3-167 MATa/MATα +pIL37-URA3*	cer	2 preferred *S. cerevisiae* alleles at *PHO84* (empty plasmid control for YMD3351)	
YMD3353		*HOΔ*::HYGb/*HOΔ*::kanMX *lys2-1*/+ *ura3Δ*::cloNAT/*ura3-167 PHO84Δ*::kanMX/+ *MATa/MATα +pIL37-PHO84-URA3*	hybrid	2+ preferred *S. cerevisiae* alleles at *PHO84*	
YMD3354		*HOΔ*::HYGb/*HOΔ*::kanMX *lys2-1*/+ *ura3Δ*::cloNAT/*ura3-167 PHO84Δ*::kanMX/+ *MATa/MATα +pIL37-URA3*	hybrid	1 preferred *S. cerevisiae* allele (empty plasmid control for YMD3353)	
YMD3355		*HOΔ*::HYGb/*HOΔ*::kanMX *lys2-1*/+ *ura3Δ*::cloNAT/*ura3-167 MATa/MATα +pIL37-PHO84-URA3*	hybrid	2+ preferred *S. cerevisiae*, 1 *S. uvarum* alleles at *PHO84*	
YMD3356		*HOΔ*::HYGb/*HOΔ*::kanMX *lys2-1*/+ *ura3Δ*::cloNAT/*ura3-167 MATa/MATα +pIL37-URA3*	hybrid	1 preferred *S. cerevisiae*, 1 *S. uvarum* allele at *PHO84* (empty plasmid control for YMD3355)	
YMD3357		*HOΔ*::natMX-GFP/+ *lys2-1*/+ +/*ura3-167 MATa/MATα*	hybrid	GFP competitor strain for hybrids	
YMD3358		*HOΔ*::natMX-GFP/+ *lys2-1*/+ +/*ura3-167 MATa/MATα* (high Ty)	hybrid	GFP competitor strain for hybrids (high Ty)	
YMD1459		*HOΔ*::kanMX-GFP/+ *MATa/MATα*	cer	GFP competitor strain for *S. cerevisiae*	

Clones are identified by nutrient (G: glucose-limitation, P: phosphate-limitation, and S: sulfate-limitation), by species ("h" denotes a hybrid, "c" denotes S. cerevisiae, "u" denotes S. uvarum), and the number indicates its derivation from independent populations. The strain number in the Dunham lab strain database is listed for each.

Supplemental Table 2: Sequencing coverage

Strain	Clone (see Table 1, 2)	Average Coverage
YMD139		58.44
YMD140		174.1
YMD129		36.6
YMD130		84.24
YMD366		176.7
YMD35	Pu1	288.3
YMD92	Pu2	60.2
YMD18	Pc3	389.8
YMD77	Pc1	82.0
YMD66	Pc4	57.1
YMD98	Pc2	100.5
YMD30	Ph1	32.4/28.15
YMD39	Ph2	39.9/34.1
YMD90	Ph3	38.4/32.4
YMD101	Ph5	19.13/17.8
YMD741	Ph4	63.2/57.5
YMD744	Ph6	37.4/69.4
YMD723	Gu1	94.3
YMD725	Gu2	80.5
YMD34	Gc2	72.2
YMD23	Gc1	104.0
YMD726	Gh1	25.6/47.8
YMD737	Gh2	35.4/30.9
YMD739	Gh3	21.2
YMD720	Su1	43.6
YMD721	Su2	74.3
YMD31	Sc3	77.4
YMD83	Sc2	90.9
YMD105	Sc1	81.6
YMD21	Sc4	133.7
YMD751	Sh2	59.8
YMD752	Sh3	31.7/29.4
YMD754	Sh4	33.7/33.0
YMD747	Sh1	100.1
YMD749	Sh7	27.6
YMD858	Sh5	33.5/34.9
YMD861	Sh6	39.2/35.1

Mean sequencing coverage is reported from GATK DepthOfCoverage for each evolved clone and ancestor.

Primer name	Primer sequence	Purpose
Sc*PHO84_URA3*homF	CGGAGACAATCATATGGGAGAAGCAATTGGAAGATAGAAAAAGGTACTCGGTACATAAA GTAACCTGACAGTATTTATA	Replace *S. cerevisiae PHO84* with *K. lactis URA3*
Sc*PHO84_URA3*homR	TGGAGGAAGTTTGAGAGGGCTTATCGCAATGGTTGTAATGGTTTAAAACTCTTTTTCGAT CCATATTATTATGAAAAGAC	Replace *S. cerevisiae PHO84* with *K. lactis URA3*
SCER_chr13_23602_*PHO84*F	TGTCCCACAGGTGCCATTGGTTG	Confirm loss of *S. cerevisiae PHO84*, gain of *URA3*
SCER_chr13_24217_*PHO84*R	CGCCTTATTCATGTTGTTGGGTATCTTCAC	Confirm loss of *S. cerevisiae PHO84*
SCER_*URA3*gain_5primeR	GGCCCAATCACAACCACATCTTAGATAGTTG	Confirm gain of *K. lactis URA3*
SCER_chr13_26112_*PHO84*F	GCCTTTCACACGACATTTGGTGCATAAC	Confirm loss of *S. cerevisiae PHO84*
SCER_chr13_26652_*PHO84*R	GACTTCAAACGACTCGGTATACTCTGCC	Confirm loss of *S. cerevisiae PHO84*, gain of *URA3*
SCER_*URA3*gain_3primeF	GAAATGCTGGATGGGAAGCGTACC	Confirm gain of *K. lactis URA3*
SCER_*PHO84*amp_chr13_23928F	GTAACCTGACAGTATTTATAGATTATGTAAGGGGTTT	Amplify the *S. cerevisiae PHO84* allele
SCER_*PHO84*amp_chr13_26478R	CCATATTATTATGAAAAGACACAACCGGAAGGGG	Amplify the *S. cerevisiae PHO84* allele
SUVA_*PHO84*amp_SCERhomF	ACAGGATGGCAGAGAGATGTGAGGAAATAATGAAATTAAAAGAAATTATCGAATAAATAT GTATAATAAATATGTAGCCT	Replace *S. cerevisiae PHO84* with *S. uvarum PHO84*
SUVA_*PHO84*amp_SCERhomR	GAAACTAATTTATCAGCCGCTCGTTTATCAACCGTTATACCAAATTATGAATAAAAAAA CTATAGAGCAGAATAATAGA	Replace *S. cerevisiae PHO84* with *S. uvarum PHO84*
SUVA_loss*PHO84*_5primeF	CGCAATCAAACATACCGTTGATACTGATGAC	Confirm loss of *S. uvarum PHO84*
SUVA_new_loss*PHO84*_5primeR	GAACGATGTCGAATCTTCTTCTCCATCTC	Confirm loss of *S. uvarum PHO84*
SUVA_new_loss*PHO84*_3primeF	CGTGGGCTATAACGACGCTACTG	Confirm loss of *S. uvarum PHO84*
SUVA_loss*PHO84*_3primeR	ATCACCAATCACGTGATCTAGTTTACCC	Confirm loss of *S. uvarum PHO84*
SUVA_*PHO84*ampF	GTATAATAAATATGTAGCCTGACAGTATTTATAGATTATGTACGT	Amplify the *S. uvarum PHO84* allele
SUVA_*PHO84*ampR	CTATAGAGCAGAATAATAGACACAACCGGCG	Amplify the *S. uvarum PHO84* allele
Suva_*PHO84*_KANMX_F	AGTTTCTCATTGAAACTTTTTTGAAATTGATTCAGTAAGC CGACATGGAGGCCCAGAATA	Replace *S. uvarum PHO84* with kanMX
Suva_*PHO84*_KANMX_R	CAGTAAGCCACAACAAACAAACAACAAGAATACATCAAAG CAGTATAGCGACCAGCATTC	Replace *S. uvarum PHO84* with kanMX
pIL37_cer*PHO84*F	GCCCGGGGGATCTCGTAACCTGACAGTATTTATAGATTATG	Cloning *S. cerevisiae PHO84* into pIL37 plasmid
pIL37_cer*PHO84*R	GAACTAGTGGATCTCGCCATATTATTATGAAAAGACACAAC	Cloning *S. cerevisiae PHO84* into pIL37 plasmid
IL429	GTGAGCGCGCGTAATACGACTC	Confirm insertion in pIL37 plasmid
IL430	CACACAGGAAACAGCTATGACCATG	Confirm insertion in pIL37 plasmid
30_chrIV_1055864F	CAAGAGGCAGTGGCAGACTTGC	Confirm SNP in evolved hybrid

30_chrIV_1055864R	CCGTGGAACGGGATTTGGCAG	Confirm SNP in evolved hybrid
39_chrV_432778F	GGACTCACAACCAGTTGACGTTG	Confirm SNP in evolved hybrid
39_chrV_432778R	CGGCTATCTCGACATATGAGTAGGC	Confirm SNP in evolved hybrid
741_chrVII_555885F	GGATGGGTTGTCAGTAGACGGTG	Confirm SNP in evolved hybrid
741_chrVII_555885F	GTCGCCCTATGCCTGGATTG	Confirm SNP in evolved hybrid
741_chrX_246208F	GTTGGTATTCAAGAATGGCGCACC	Confirm SNP in evolved hybrid
741_chrX_246208R	GACTCCGAGTGAGCTTCGACATC	Confirm SNP in evolved hybrid
744_chrVII_972813F	GCGGTGGCATCAATGTAGTCAACC	Confirm SNP in evolved hybrid
744_chrVII_972813R	GATTCTCCAGGTATGAATGCCGCTG	Confirm SNP in evolved hybrid
726_chrXIII_852028F	CGGGTAACGAAGAATCGACTCTCGC	Confirm SNP in evolved hybrid
726_chrXIII_852028R	GCGGAATAACCTAACACGTGGAGG	Confirm SNP in evolved hybrid
737_chrIV_111919F	GATACGACAGGTAGCATACGAACCCC	Confirm SNP in evolved hybrid
737_chrIV_111919R	GTGATTCCTCCAGCACCTACCTG	Confirm SNP in evolved hybrid
737_chrIII_51593F	GGATGTCGTGCAATTGTACCAGGAG	Confirm SNP in evolved hybrid
737_chrIII_51593R	GCTTGAGCTCATTATCGAAGGACCC	Confirm SNP in evolved hybrid
749_chrII_238875F	GCAAGAAGATCTGGCAAGCTACAAGG	Confirm SNP in evolved hybrid
749_chrII_238875R	CGTCGCTACAGAAGGCTTTGGTTG	Confirm SNP in evolved hybrid
749_chrXVI_490631F	CCAATGCAGGTGGCTTATTGGTTG	Confirm SNP in evolved hybrid
749_chrXVI_490631R	GGCGAAAGTAAGTCCAGCACTGAG	Confirm SNP in evolved hybrid
749_chrXVI_86106F	CTGGTAGTCAACCATCCCGAGATG	Confirm SNP in evolved hybrid
749_chrXVI_86106R	GGGCAGCAACCCTAAACTGCTG	Confirm SNP in evolved hybrid
751_chrVII_936384F	CATGCTCGTTTCCCATATGACCAGG	Confirm SNP in evolved hybrid
751_chrVII_936384R	GCATAGAACTTCGGAGGTCAATGGG	Confirm SNP in evolved hybrid
752_chrVI_162998F	GGAATTGACCGGTGATACGTCCAAG	Confirm SNP in evolved hybrid
752_chrVI_162998R	GCCCACAAGACCATATCTGTGACC	Confirm SNP in evolved hybrid
754_chrXIV_666675F	GAATTGAGCTCTCAGTCTTTAAGCGACC	Confirm SNP in evolved hybrid
754_chrXIV_666675R	CTGGGACGATATCAACGAGCAATCC	Confirm SNP in evolved hybrid
754_chrXV_800832F	CTGCTGTTGGCACTGATATGCC	Confirm SNP in evolved hybrid
754_chrXV_800832R	CCCAGCAAGGCGATGATTCTAACTTC	Confirm SNP in evolved hybrid
754_chrIV_259517F	CCTTAGCCTGCTTCACTACTTAGAAGTTG	Confirm SNP in evolved hybrid
754_chrIV_259517R	CCAGCTCTGTTTGAATGTGCCTCC	Confirm SNP in evolved hybrid
861_chrV_269392F	TACGTCGTCTACAGTTGGACAGGC	Confirm SNP in evolved hybrid
861_chrV_269392R	GTGGTTCCAGATGTGCCAACTGTG	Confirm SNP in evolved hybrid
861_chrXIV_746688F	AACTGGTCTTTGGCAAATGGCTCC	Confirm SNP in evolved hybrid
861_chrXIV_746688R	CAGACACAGTTTTTCAGCGATGCCC	Confirm SNP in evolved hybrid
39_uva_chrVII_9524F	GGCGCCTAATGTGGAAGAAGATGAC	Confirm SNP in evolved hybrid
39_uva_chrVII_9524R	GTCTTGTATGCTGGTAGGCAGGTG	Confirm SNP in evolved hybrid
39_uva_chrXVI_232879F	CTAAGACCGTTCCCGTTCCTAAGC	Confirm SNP in evolved hybrid
39_uva_chrXVI_232879R	GCCACCAGTTTAGCTATATCTTTCGCTG	Confirm SNP in evolved hybrid
39_uva_chrXIII_194496F	GATTCACCACGCCTAAGCTCTTGAG	Confirm SNP in evolved hybrid
39_uva_chrXIII_194496R	GTTGTCAAGGAATTGCCAAAGCC	Confirm SNP in evolved hybrid

39_uva_chrIV_244399F	GAACCGACCTAGCTTCAACCCTTC	Confirm SNP in evolved hybrid
39_uva_chrIV_244399R	ATACGATGTCAGCCTCGCAGGAG	Confirm SNP in evolved hybrid
101_uva_chrXIII_231731F	GGATCATCGAAGGCGGTAGATCG	Confirm SNP in evolved hybrid
101_uva_chrXIII_231731R	CACAAACCCTCCTGCACACCTTTC	Confirm SNP in evolved hybrid
741_uva_chrXIII_324121F	GTCCATAGCACGCTGTGCTTCTG	Confirm SNP in evolved hybrid
741_uva_chrXIII_324121R	GAGCATGGTAGACTGAACCAGTTCAG	Confirm SNP in evolved hybrid
749_uva_chrII_273296F	GTGGCTTGTCCATGAGATGCAACAG	Confirm SNP in evolved hybrid
749_uva_chrII_273296R	CCACTCCGTGAGACCCAATCTTC	Confirm SNP in evolved hybrid
752_uva_chrXIV_495890F	GTGACGGTACATGTGGTACGAACC	Confirm SNP in evolved hybrid
752_uva_chrXIV_495890R	CGCGACTATGATTACACAGGCCATAC	Confirm SNP in evolved hybrid
754_uva_chrV_342563F	GCCAATTCGGTACTTCCTTCTGAGC	Confirm SNP in evolved hybrid
754_uva_chrV_342563R	GAATCGAGTGCCTAGCGAGAGC	Confirm SNP in evolved hybrid
754_uva_chrX_769768F	CTGTCCTGGAGACTTTCAGATTCGG	Confirm SNP in evolved hybrid
754_uva_chrX_769768R	GCTGTCTTCGCCTTCGTCTGTAC	Confirm SNP in evolved hybrid
754_uva_chrX_990873F	CACCATGACTGTCGCTATGTCAAACG	Confirm SNP in evolved hybrid
754_uva_chrX_990873R	CACATTTGGTACATGGTTCTGGTGCTC	Confirm SNP in evolved hybrid
754_uva_chrXII_192491F	AAAGCAGTACTCGACATGCGGC	Confirm SNP in evolved hybrid
754_uva_chrXII_192491R	CTGACAGGGATGCTGGTGACAAG	Confirm SNP in evolved hybrid
754_uva_chrXIV_19697F	CGGGTCGTTCCCTCAAGAGTC	Confirm SNP in evolved hybrid
754_uva_chrXIV_19697R	GGGACAGATTTGACAGGTTCATTGAAGG	Confirm SNP in evolved hybrid
858_uva_chrIV_310881F	CAACAGGGGAAGGTTGCCTATGAC	Confirm SNP in evolved hybrid
858_uva_chrIV_310881R	CAATGTCGGTAAGTGAACAGGACCC	Confirm SNP in evolved hybrid
858_uva_chrVIII_16911F	GCCTCCTTCATAGCCCTGCTTTC	Confirm SNP in evolved hybrid
858_uva_chrVIII_16911R	CCCGAATCCACCCTCTTACCATAC	Confirm SNP in evolved hybrid
861_uva_chrIV_413046F	GTTAGACCGATCCAGCAGGAGC	Confirm SNP in evolved hybrid
861_uva_chrIV_413046R	GAACCTATTCCTAAGCAGGGTCCG	Confirm SNP in evolved hybrid
739_uva_chrII_889421F	GAAGGTCGAGGCAGATCTATCCACC	Confirm SNP in evolved hybrid
739_uva_chrII_889421R	GCGTAGGTGCAGAAACCACCC	Confirm SNP in evolved hybrid

Supplemental Table 3: Primers used

Primers used for allele replacements and SNP confirmation in evolved hybrid clones.

Supplementary Figure 1

A

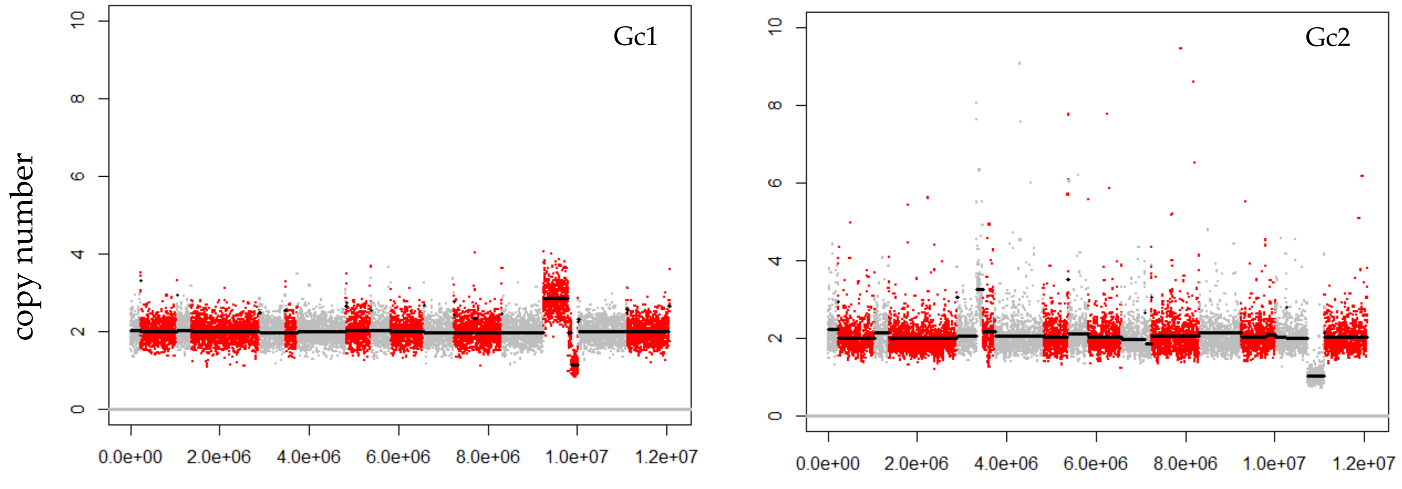

B

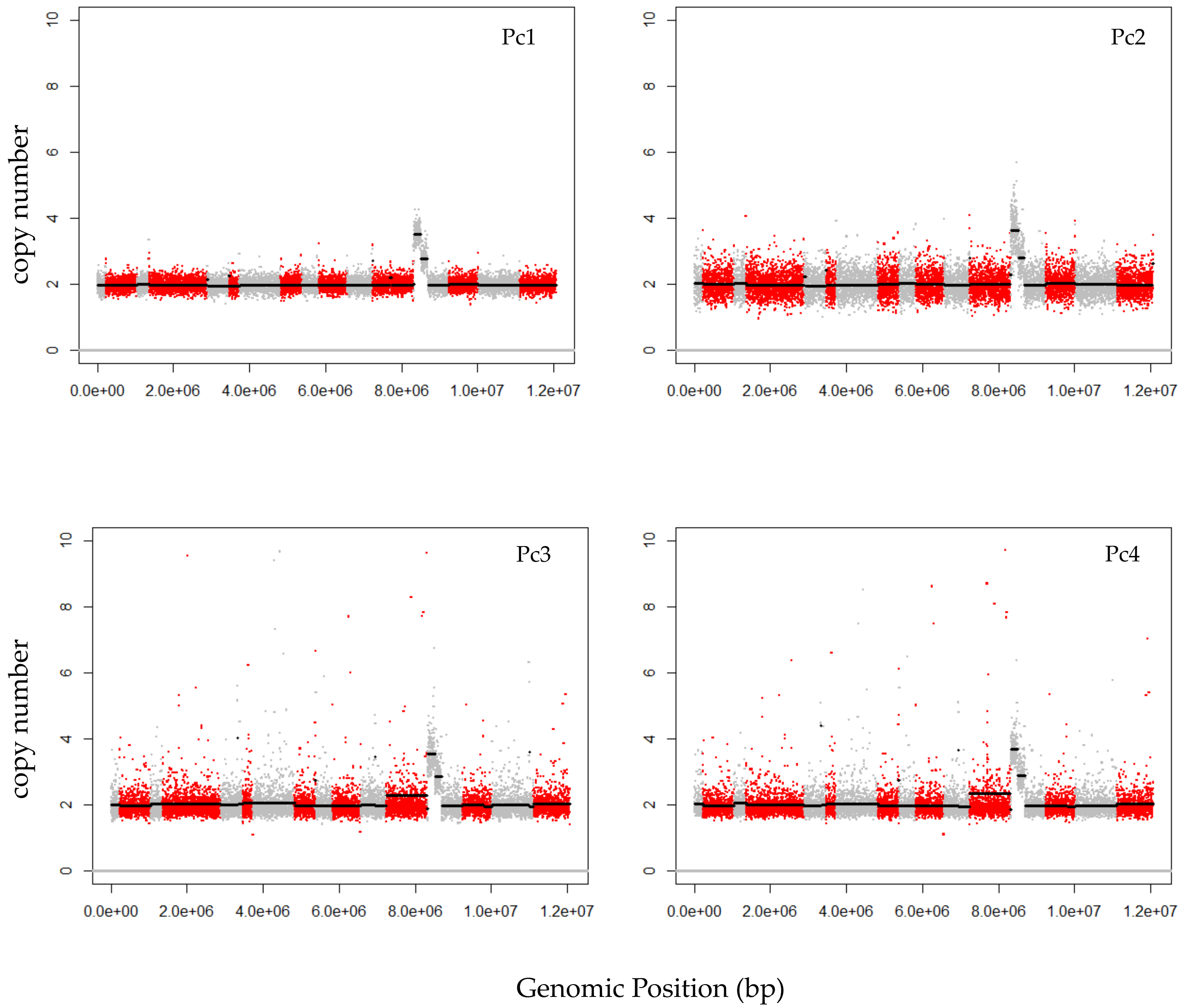

C

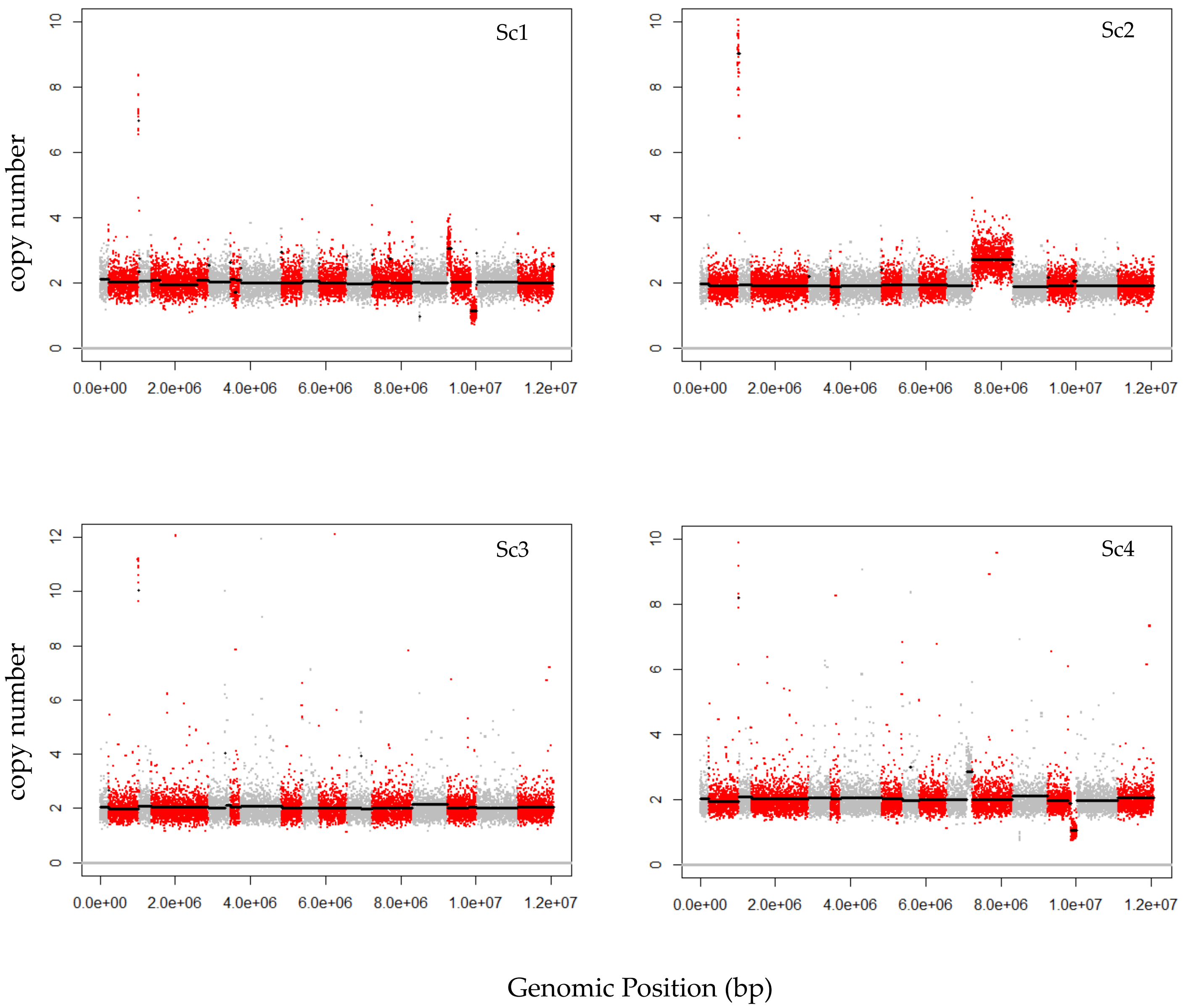
Sc1
Sc2
Sc3
Sc4
copy number
copy number
0
2
4
6
8
10
12
0.0e+00
2.0e+06
4.0e+06
6.0e+06
8.0e+06
1.0e+07
1.2e+07
Genomic Position (bp)

Supplementary Figure 2

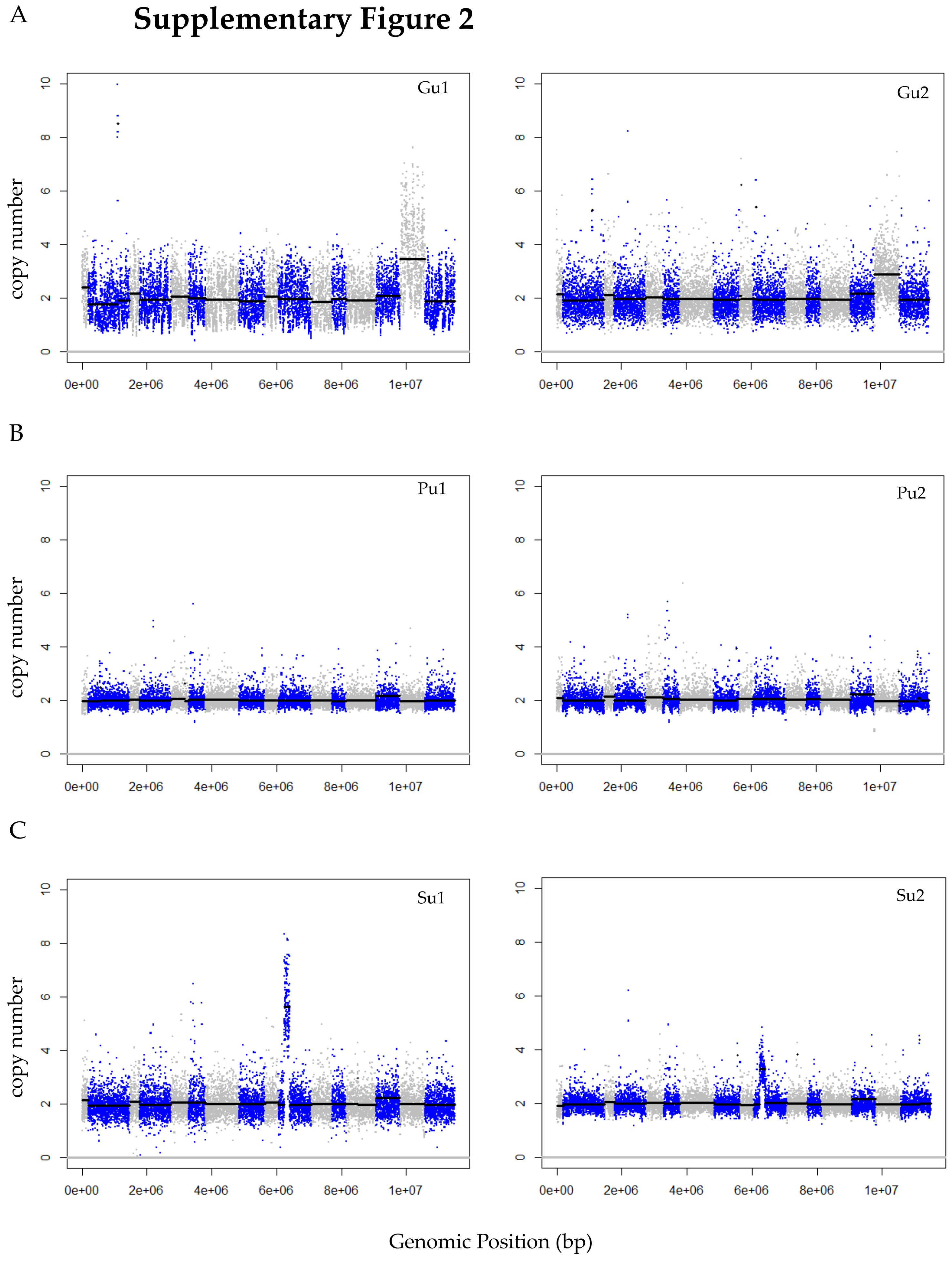

Supplementary Figure 3

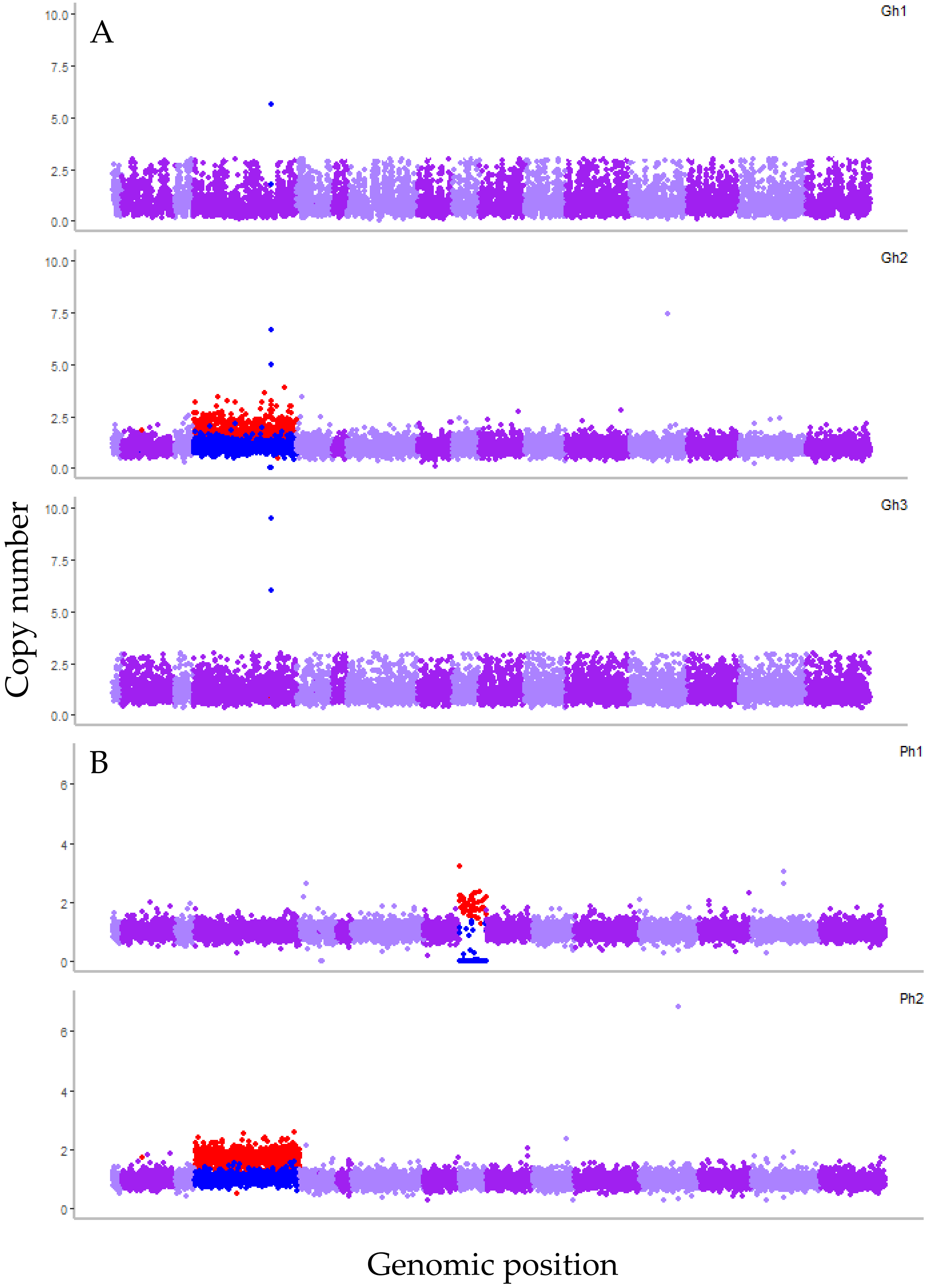

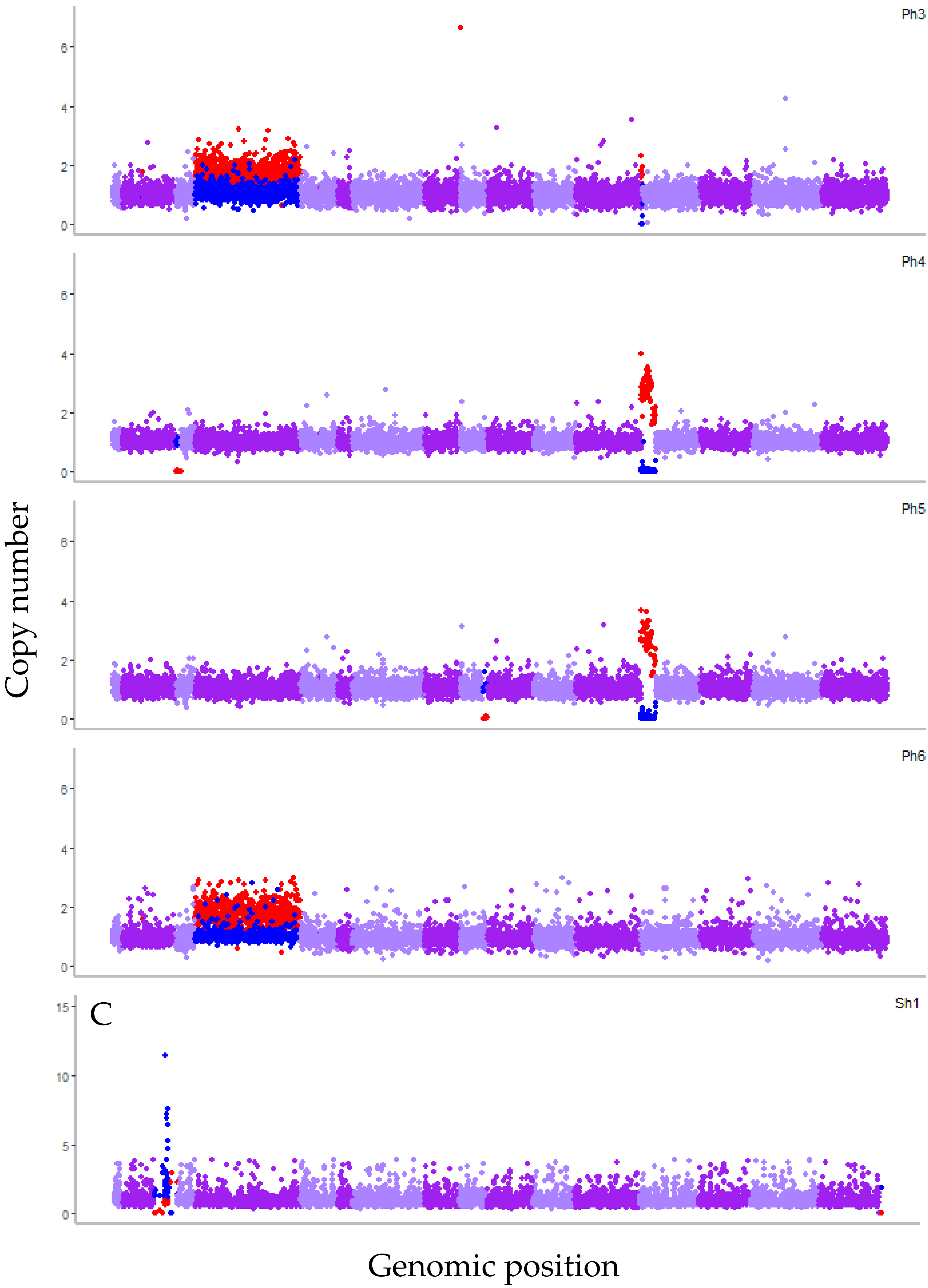
Ph3
Ph4
Ph5
Ph6
Sh1
C
0
2
4
6
5
10
15
Copy number
Genomic position

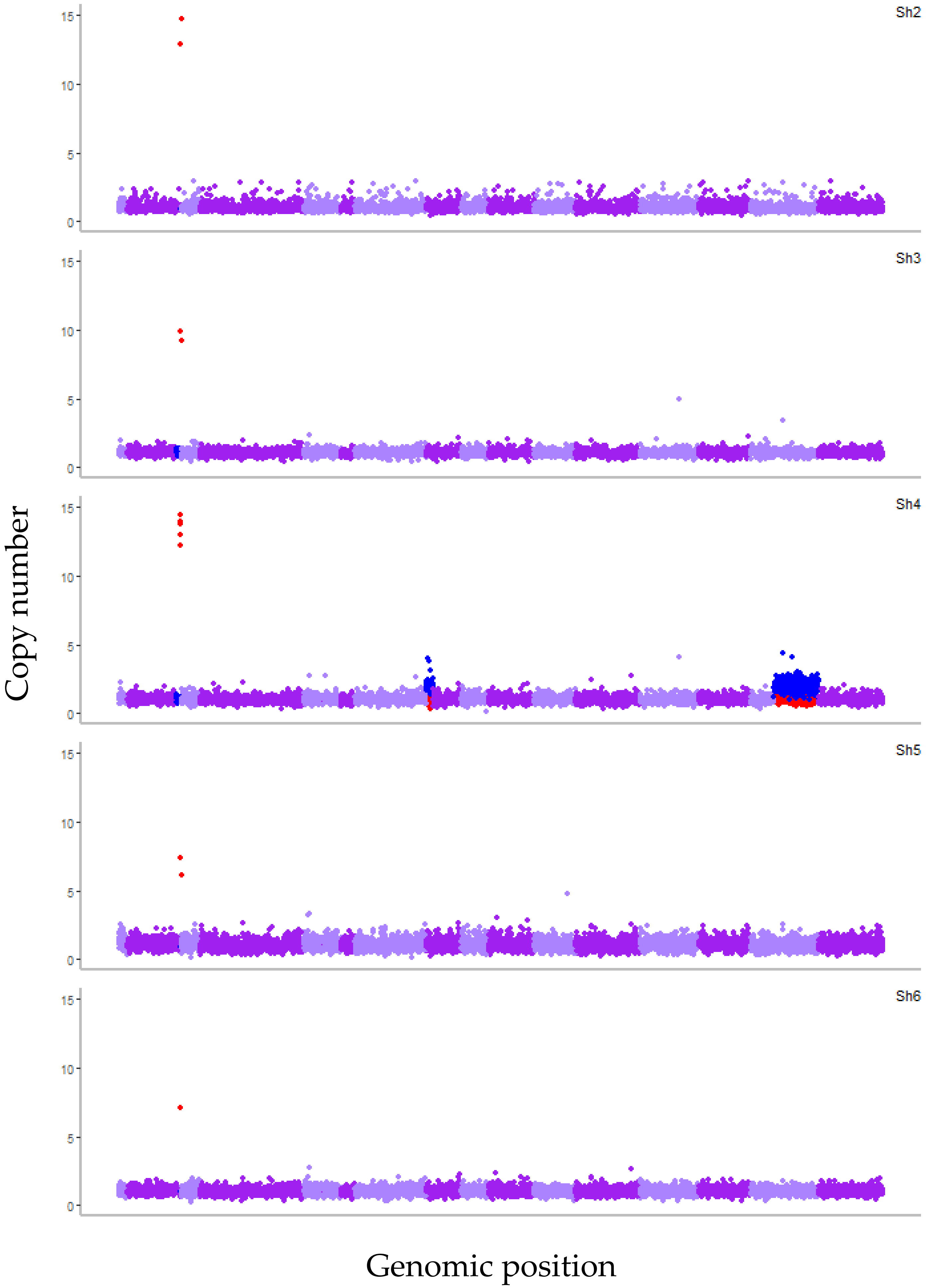

Sh2
Sh3
Sh4
Sh5
Sh6
15
10
5
0
Copy number
Genomic position

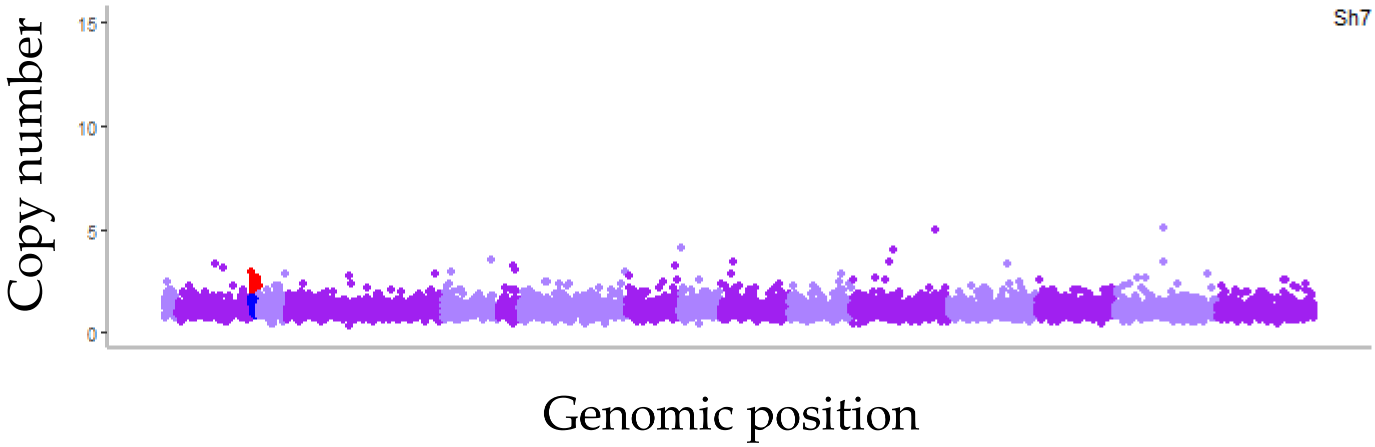

Copy number
15
10
5
0
Sh7
Genomic position

Supplementary Figure 4

A

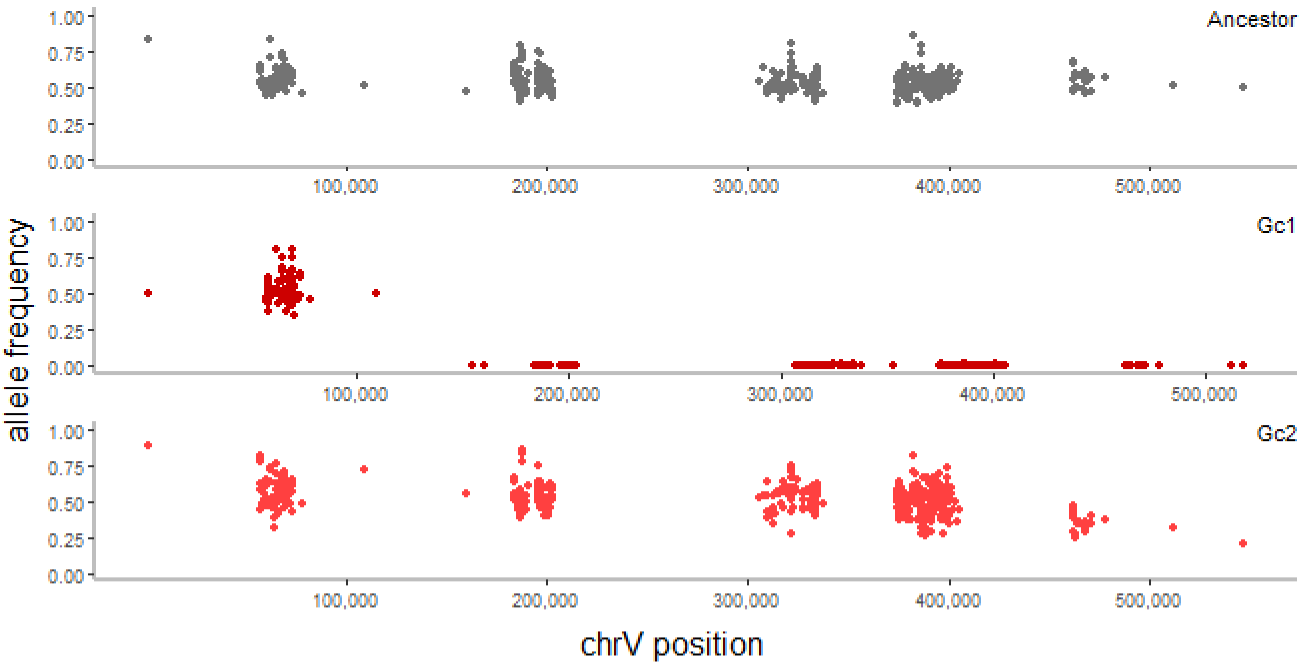

B

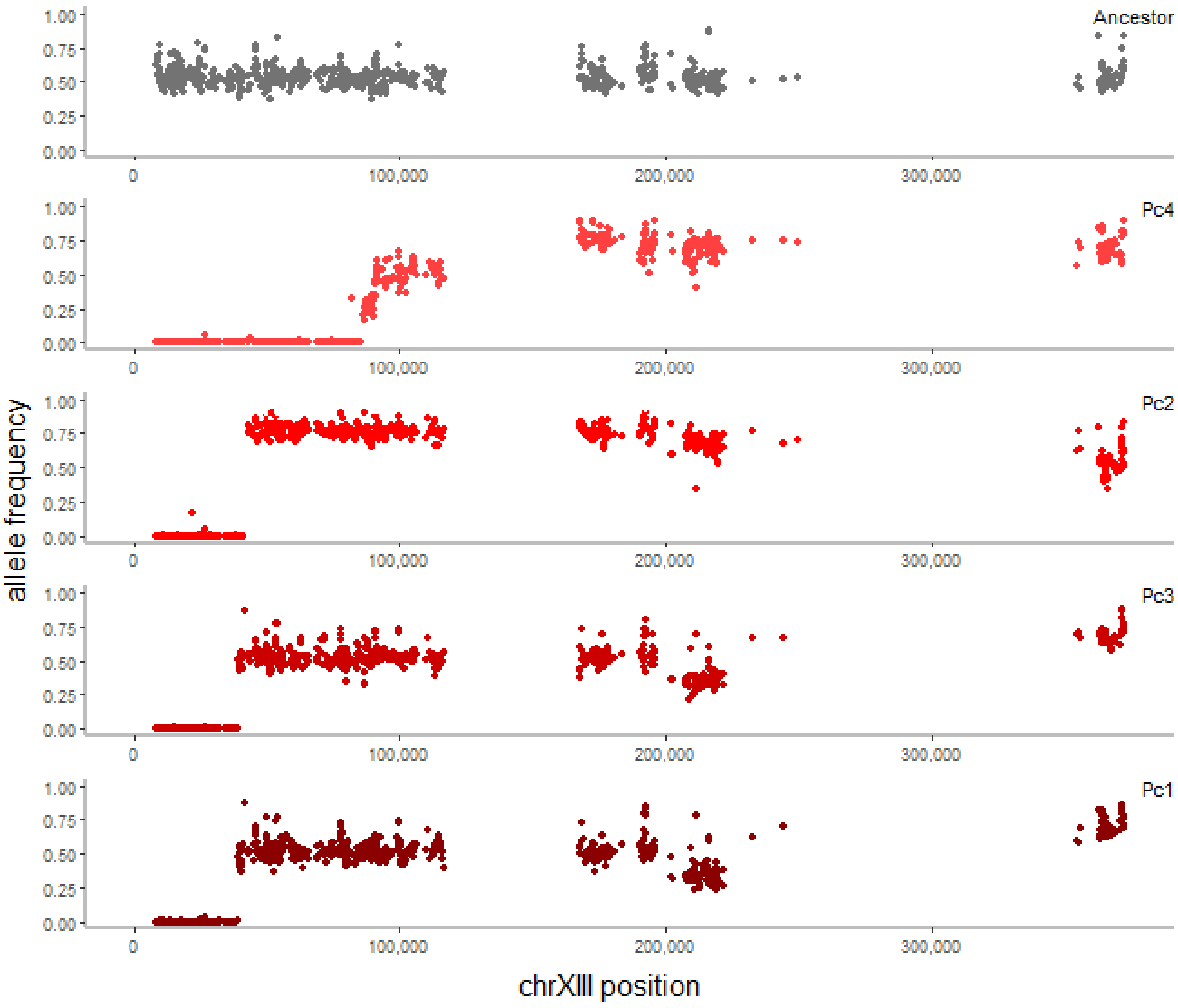

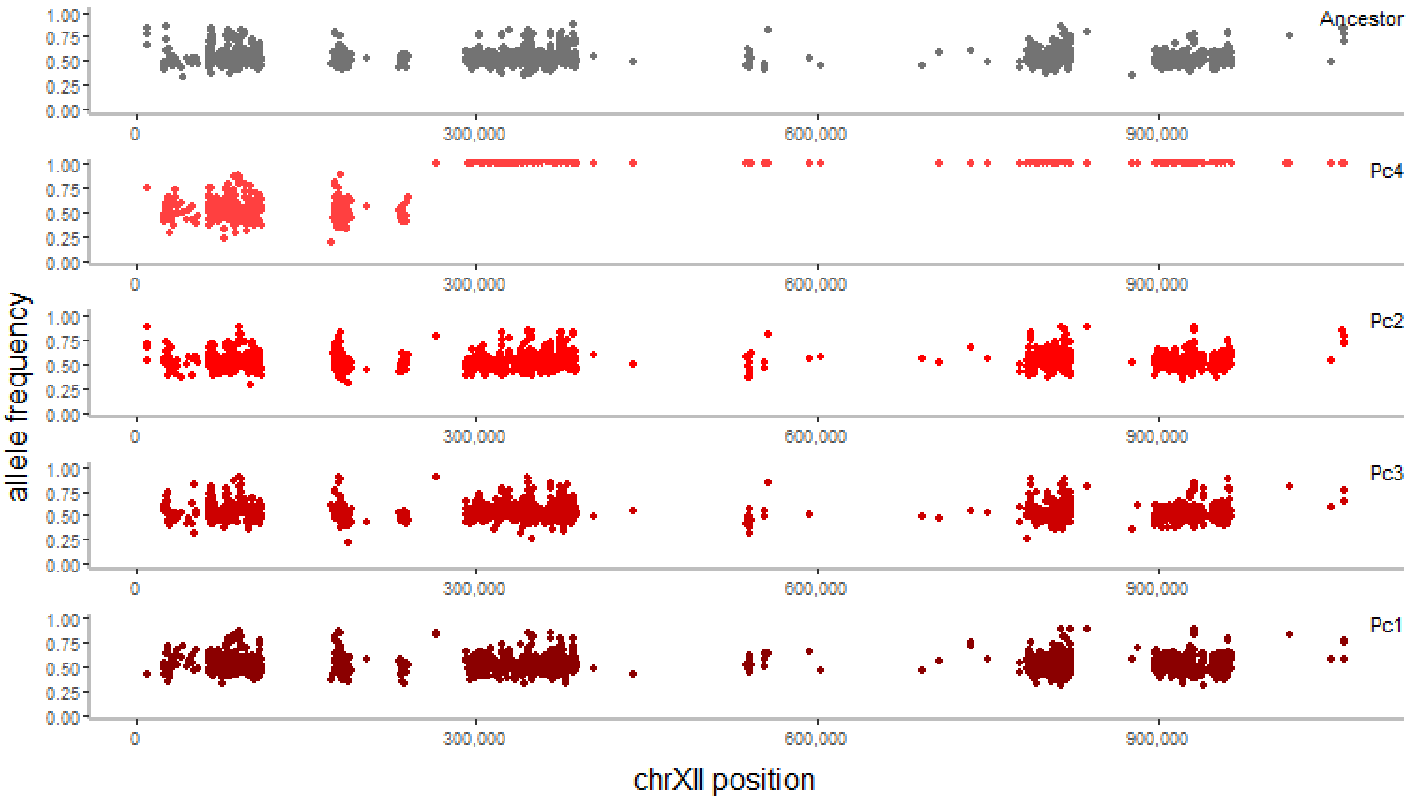
Ancestor
Pc4
Pc2
Pc3
Pc1
allele frequency
1.00
0.75
0.50
0.25
0.00
0
300,000
600,000
900,000
chrXII position

C

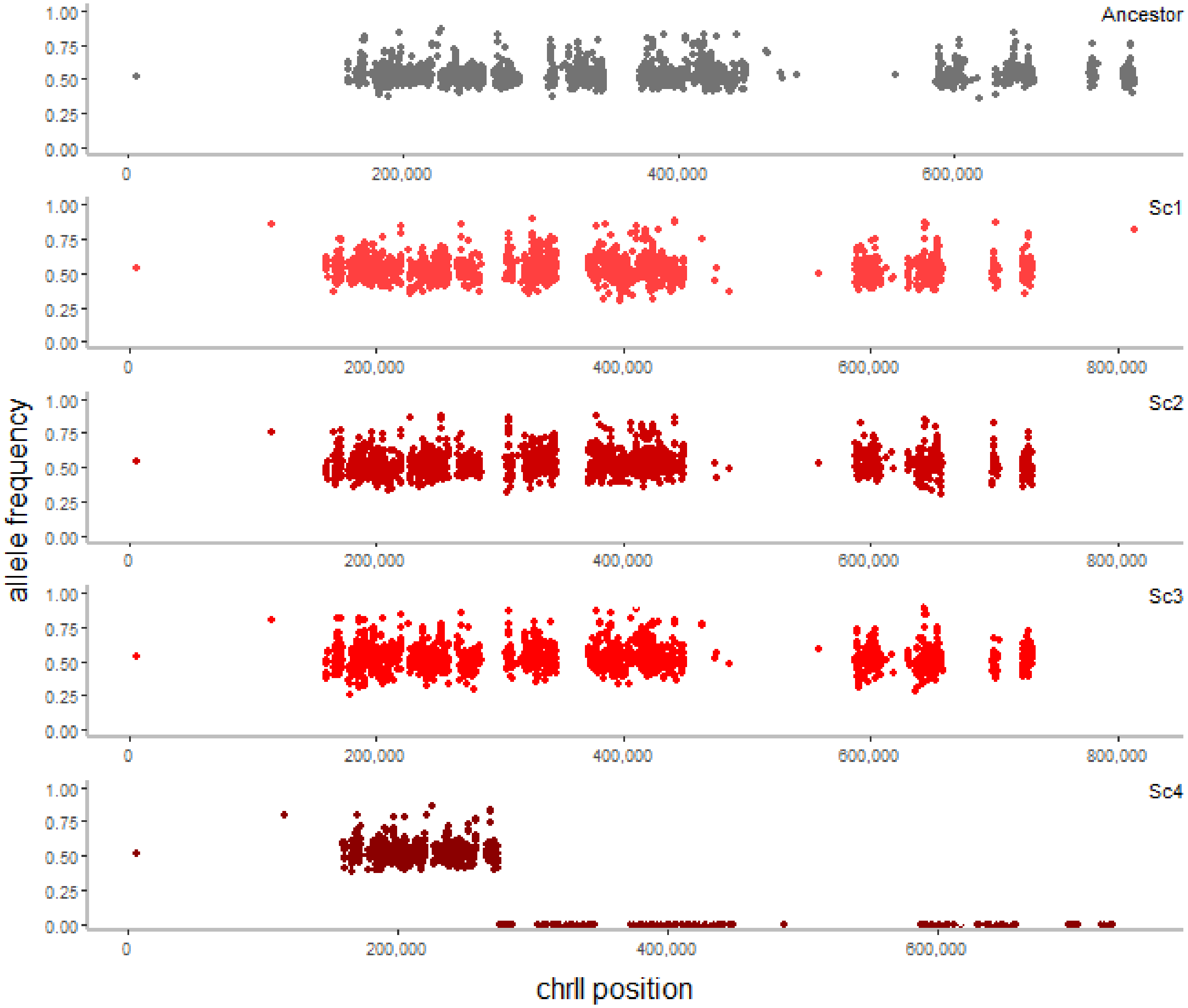
Ancestor
Sc1
Sc2
Sc3
Sc4
allele frequency
1.00
0.75
0.50
0.25
0.00
0
200,000
400,000
600,000
800,000
chrII position

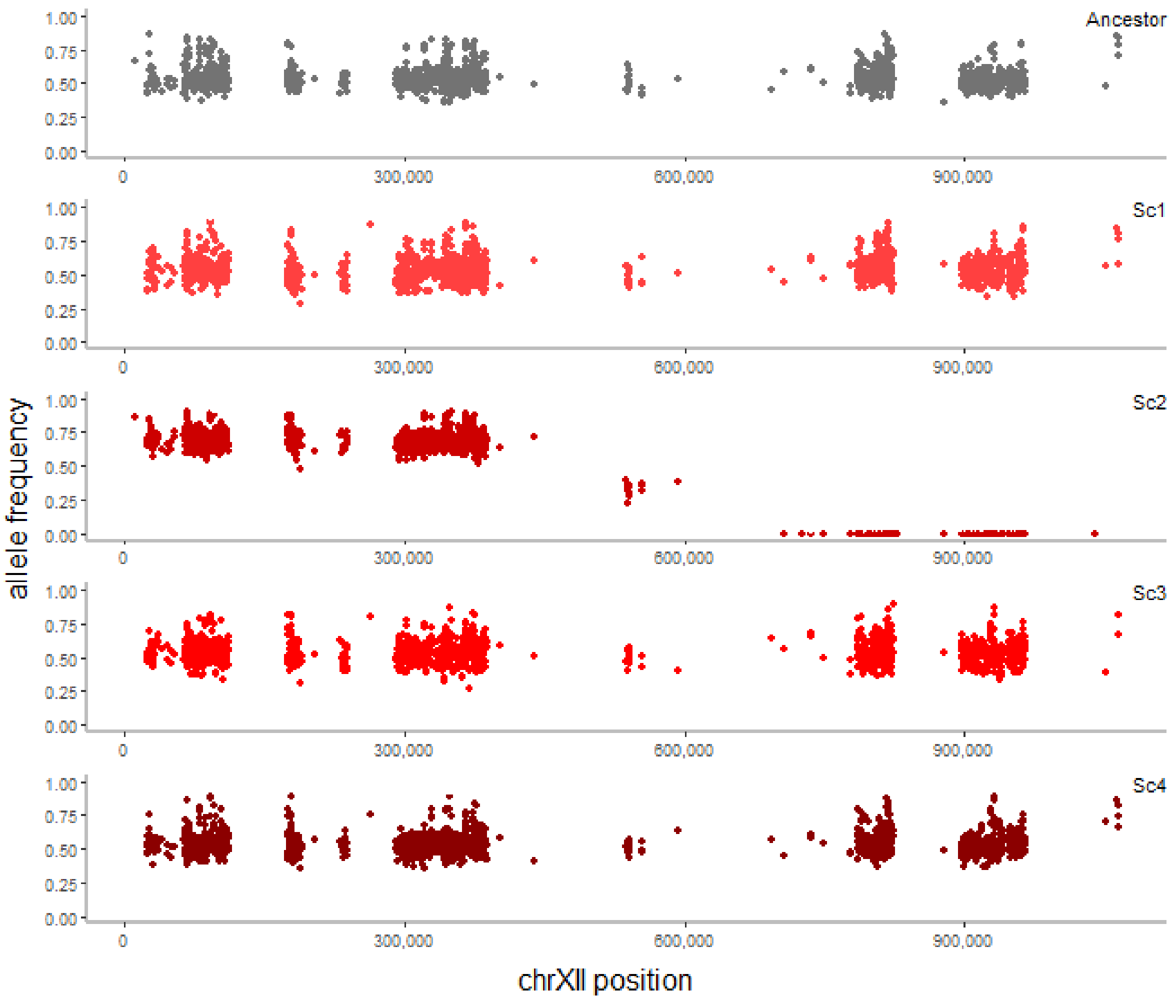
Ancestor
Sc1
Sc2
Sc3
Sc4
allele frequency
1.00
0.75
0.50
0.25
0.00
0
300,000
600,000
900,000
chrXII position

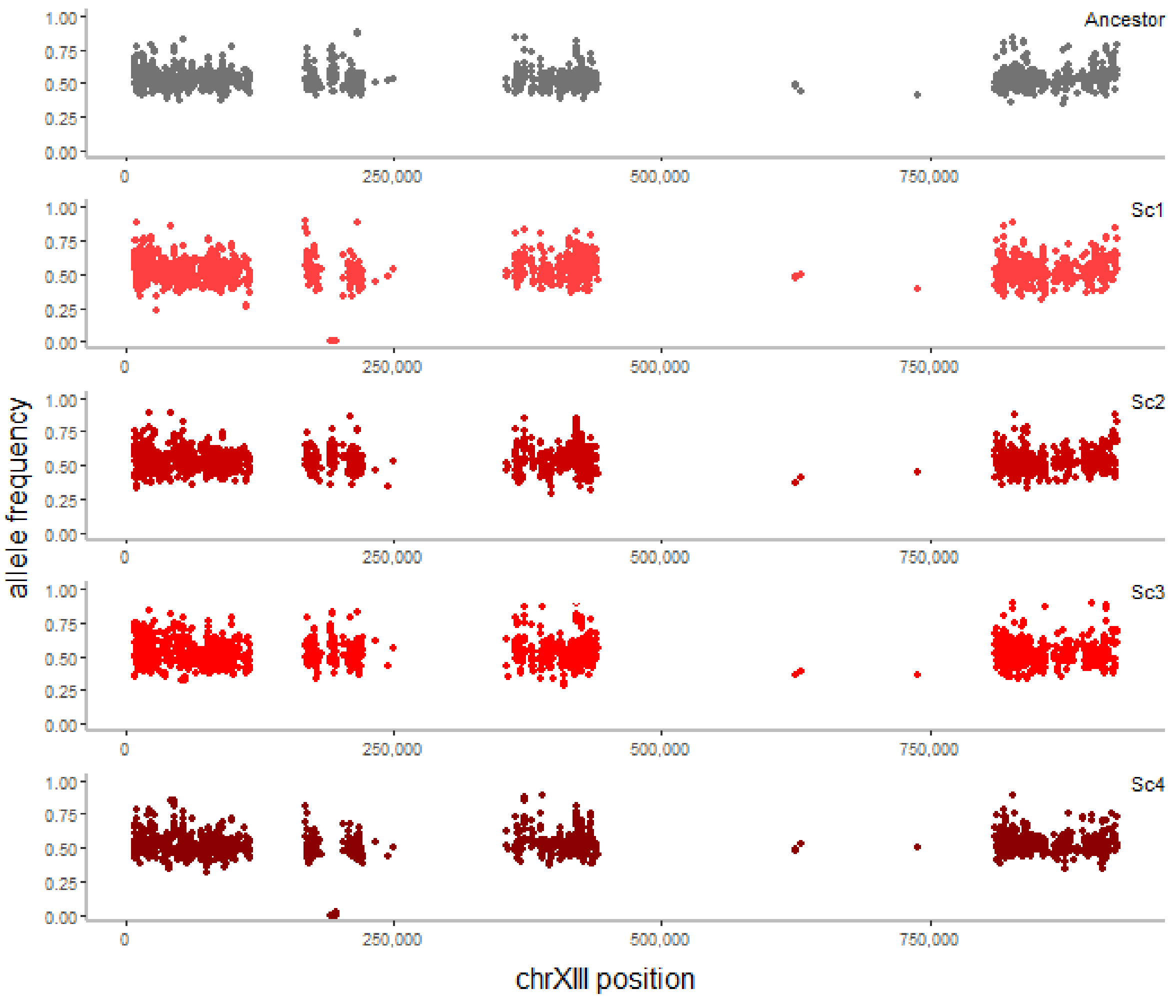
Ancestor
Sc1
Sc2
Sc3
Sc4
allele frequency
1.00
0.75
0.50
0.25
0.00
0
250,000
500,000
750,000
chrXIII position

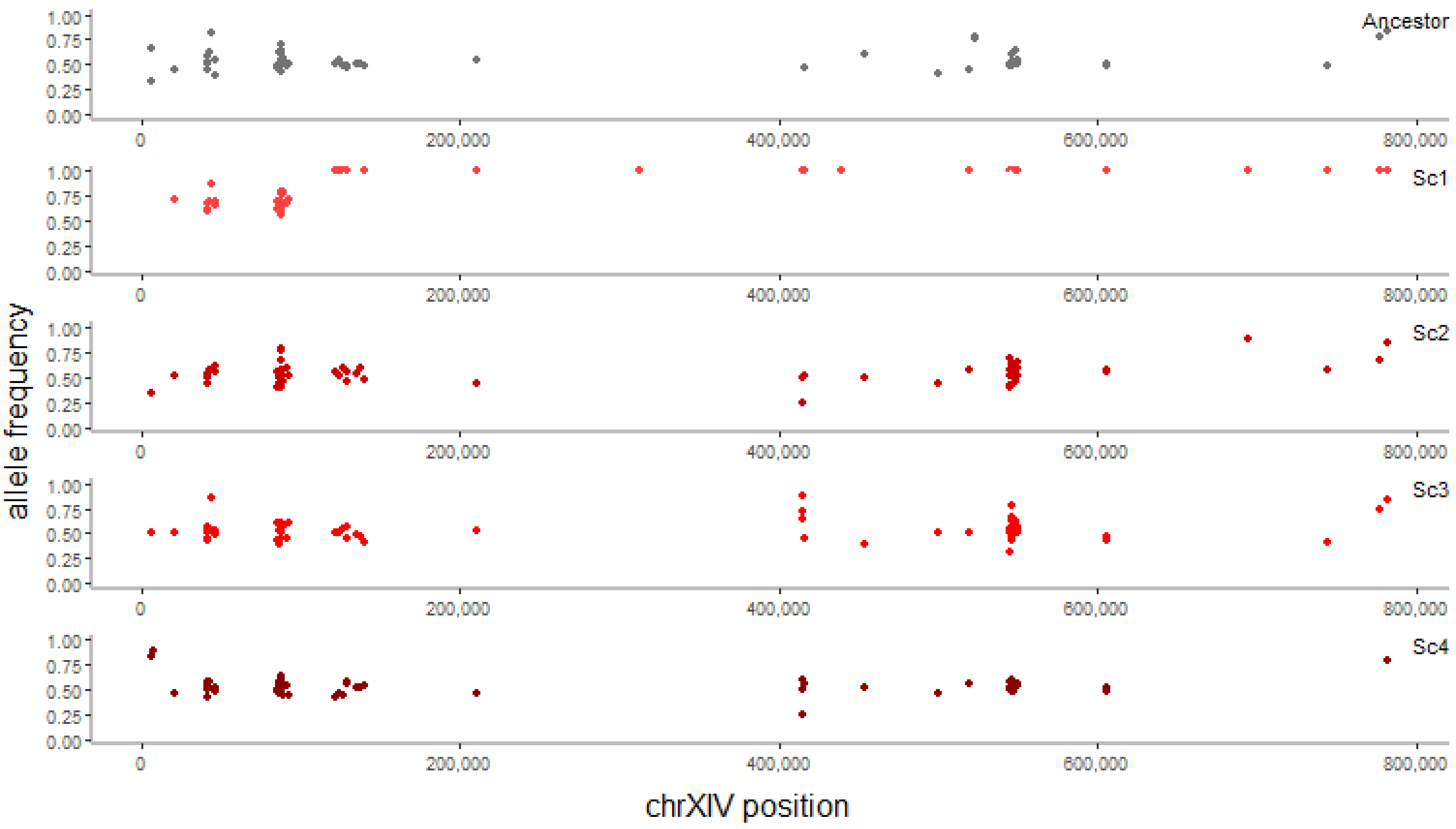
Ancestor
Sc1
Sc2
Sc3
Sc4
allele frequency
chrXIV position
0
200,000
400,000
600,000
800,000
0.00
0.25
0.50
0.75
1.00

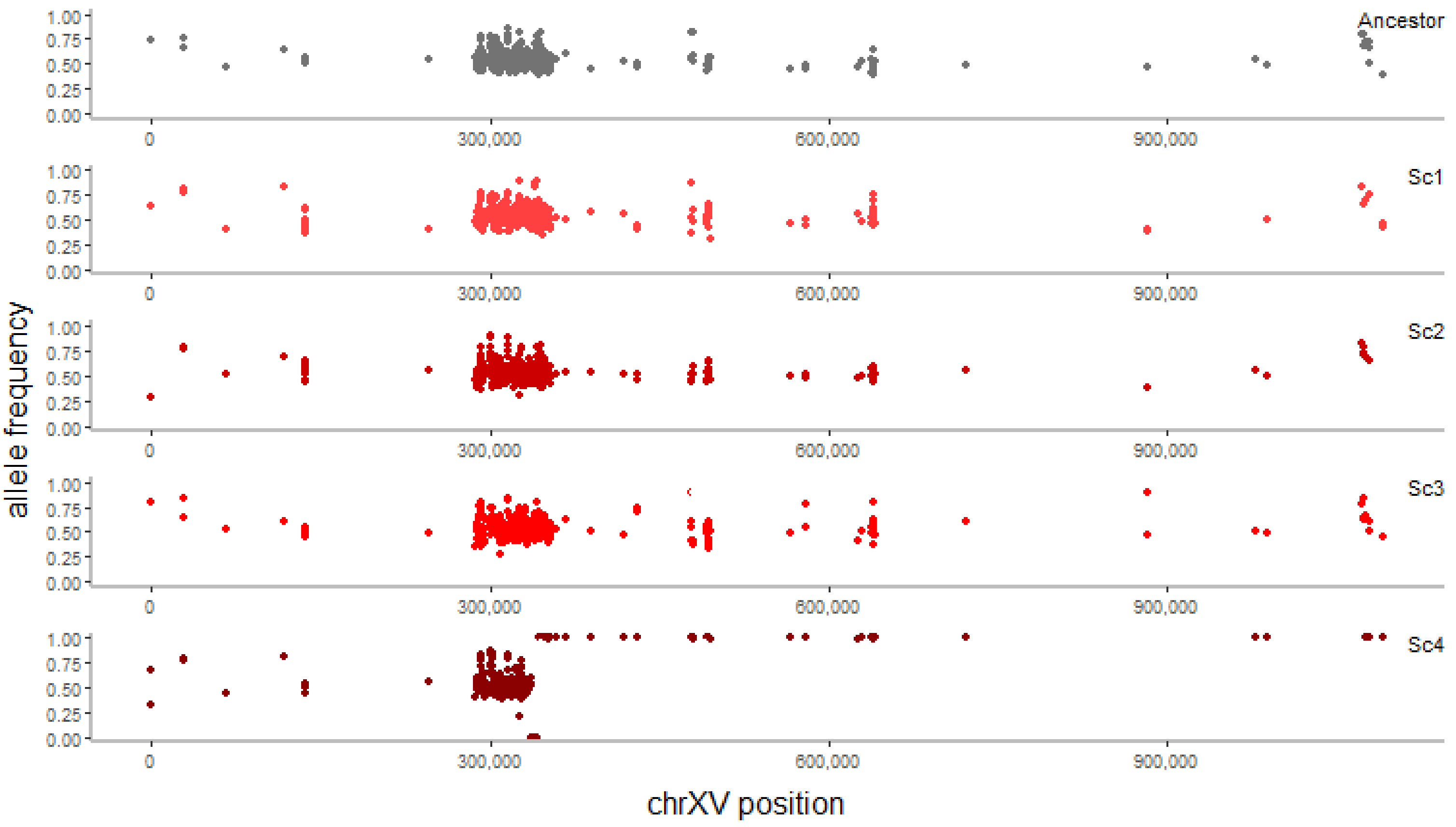
Ancestor
Sc1
Sc2
Sc3
Sc4
allele frequency
chrXV position
0
300,000
600,000
900,000
0.00
0.25
0.50
0.75
1.00

Supplementary Figure 5

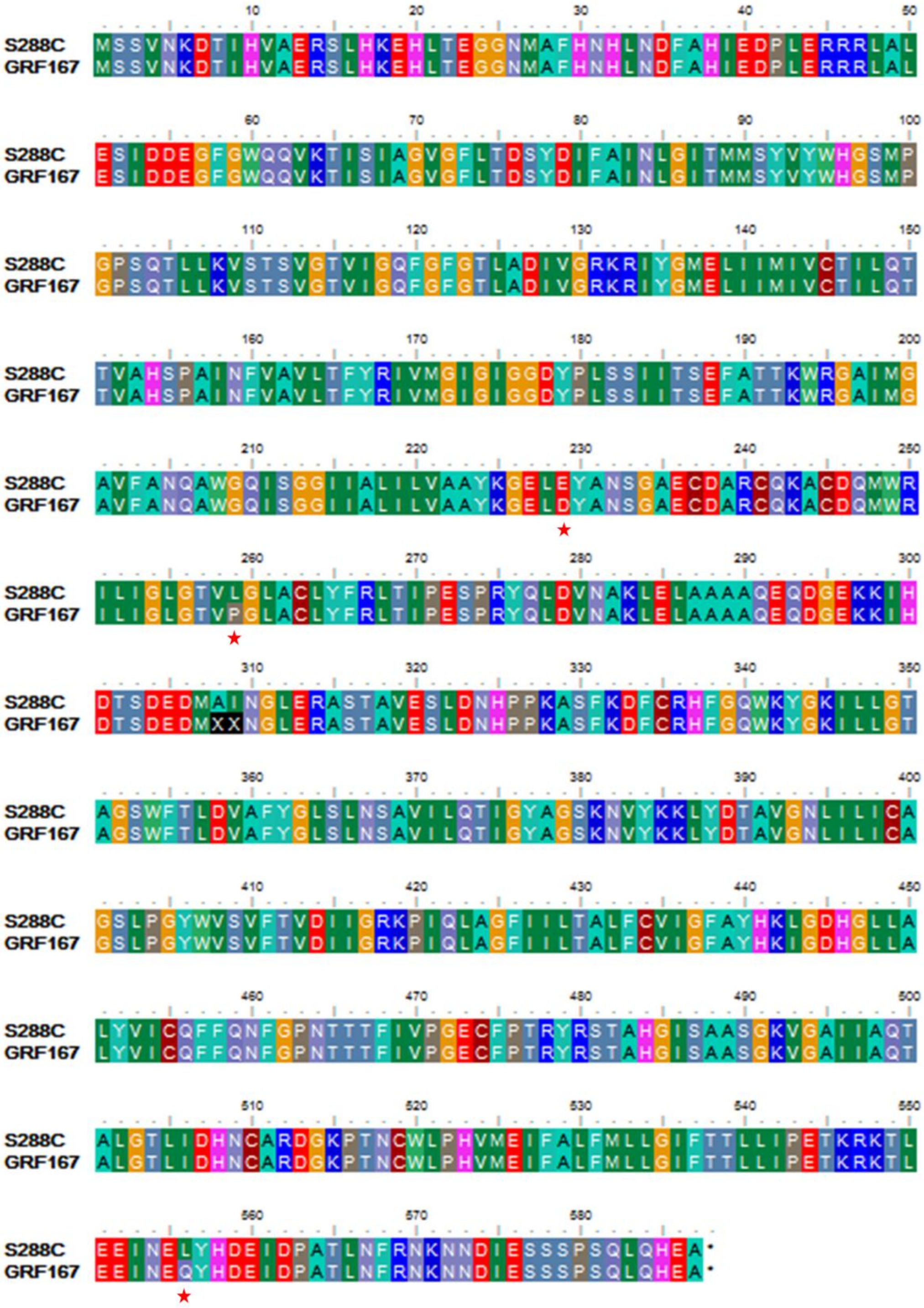

```
                10        20        30        40        50        60        70        80
        ....|....|....|....|....|....|....|....|....|....|....|....|....|....|....|....|
S288C   ATGAGTTCCGTCAATAAAGATACTATTCATGTTGCTGAAAGAAGTCTTCATAAAGAACACCTTACCGAAGGTGGTAACAT
         M  S  S  V  N  K  D  T  I  H  V  A  E  R  S  L  H  K  E  H  L  T  E  G  G  N  M
GRF167  ATGAGTTCCGTCAATAAAGATACTATTCATGTTGCTGAAAGAAGTCTTCATAAAGAACACCTTACCGAAGGTGGTAACAT
         M  S  S  V  N  K  D  T  I  H  V  A  E  R  S  L  H  K  E  H  L  T  E  G  G  N  M
UVA     ATGAGTTCCGTTGCTAAAGATAATATTAGTATTGCTGAAAGAAGTCTTCACAGGGAAAACTTGACTGAAGGTGGTAACCT
         M  S  S  V  A  K  D  N  I  S  I  A  E  R  S  L  H  R  E  N  L  T  E  G  G  N  L

                90       100       110       120       130       140       150       160
        ....|....|....|....|....|....|....|....|....|....|....|....|....|....|....|....|
S288C   GGCCTTCCACAACCATTTGAATGATTTTGCTCATATTGAAGATCCTCTGGAAAGAAGAAGATTGGCTTTGGAGTCCATCG
          A  F  H  N  H  L  N  D  F  A  H  I  E  D  P  L  E  R  R  R  L  A  L  E  S  I
GRF167  GGCCTTCCACAACCATTTGAATGATTTTGCTCATATTGAAGATCCTCTGGAAAGAAGAAGATTGGCTTTGGAGTCCATCG
          A  F  H  N  H  L  N  D  F  A  H  I  E  D  P  L  E  R  R  R  L  A  L  E  S  I
UVA     GGCGTTCCACAATCATTTGAATGATTTCGCCCATATCGAAGATCCTTTGGAAAGAAGAAGATTGGCTTTGGAATCCATTG
          A  F  H  N  H  L  N  D  F  A  H  I  E  D  P  L  E  R  R  R  L  A  L  E  S  I

               170       180       190       200       210       220       230       240
        ....|....|....|....|....|....|....|....|....|....|....|....|....|....|....|....|
S288C   ATGACGAAGGTTTCGGTTGGCAACAAGTTAAGACCATCTCCATTGCTGGTGTTGGTTTCTTGACAGATTCTTATGATATT
        D  D  E  G  F  G  W  Q  Q  V  K  T  I  S  I  A  G  V  G  F  L  T  D  S  Y  D  I
GRF167  ATGACGAAGGTTTCGGTTGGCAACAAGTTAAGACCATCTCCATTGCTGGTGTTGGTTTCTTGACAGATTCTTATGATATT
        D  D  E  G  F  G  W  Q  Q  V  K  T  I  S  I  A  G  V  G  F  L  T  D  S  Y  D  I
UVA     ATAACGAAGGTTTCGGTTGGCAACAGGTCAAGACCATCTCCATTGCCGGTGTTGGTTTCTTGACCGATTCGTACGATATC
        D  N  E  G  F  G  W  Q  Q  V  K  T  I  S  I  A  G  V  G  F  L  T  D  S  Y  D  I

               250       260       270       280       290       300       310       320
        ....|....|....|....|....|....|....|....|....|....|....|....|....|....|....|....|
S288C   TTTGCCATTAATTTGGGTATCACTATGATGTCCTACGTTTACTGGCACGGTAGTATGCCAGGTCCAAGTCAAACCTTGTT
         F  A  I  N  L  G  I  T  M  M  S  Y  V  Y  W  H  G  S  M  P  G  P  S  Q  T  L  L
GRF167  TTTGCCATTAATTTGGGTATCACTATGATGTCCTACGTTTACTGGCACGGTAGTATGCCAGGTCCAAGTCAAACCTTGTT
         F  A  I  N  L  G  I  T  M  M  S  Y  V  Y  W  H  G  S  M  P  G  P  S  Q  T  L  L
UVA     TTCGCCATCAACTTGGGTATCTCCATGATGTCCTATGTCTACTGGCACGGTGACATGCCAGCTTCCAGTCAAACCTTGTT
         F  A  I  N  L  G  I  S  M  M  S  Y  V  Y  W  H  G  D  M  P  A  S  S  Q  T  L  L

               330       340       350       360       370       380       390       400
        ....|....|....|....|....|....|....|....|....|....|....|....|....|....|....|....|
S288C   GAAGGTTTCCACTTCTGTTGGTACTGTTATTGGTCAATTTGGTTTTGGTACTTTAGCTGATATTGTTGGTCGTAAGAGAA
          K  V  S  T  S  V  G  T  V  I  G  Q  F  G  F  G  T  L  A  D  I  V  G  R  K  R
GRF167  GAAGGTTTCCACTTCTGTTGGTACTGTTATTGGTCAATTTGGTTTTGGTACTTTAGCTGATATTGTTGGTCGTAAGAGAA
          K  V  S  T  S  V  G  T  V  I  G  Q  F  G  F  G  T  L  A  D  I  V  G  R  K  R
UVA     GAAGGTTTCCACTTCTGTCGGTACTGTTATTGGTCAAGTCGGTTTCGGTACTTTGGCCGATATCGTTGGTCGTAAGAAGA
          K  V  S  T  S  V  G  T  V  I  G  Q  V  G  F  G  T  L  A  D  I  V  G  R  K  K

               410       420       430       440       450       460       470       480
        ....|....|....|....|....|....|....|....|....|....|....|....|....|....|....|....|
S288C   TTTATGGTATGGAACTTATTATCATGATTGTCTGTACCATTCTGCAAACCACTGTTGCTCATTCTCCTGCTATTAACTTC
        I  Y  G  M  E  L  I  I  M  I  V  C  T  I  L  Q  T  T  V  A  H  S  P  A  I  N  F
GRF167  TTTATGGTATGGAACTTATTATCATGATTGTCTGTACCATTCTGCAAACCACTGTTGCTCATTCTCCTGCTATTAACTTC
        I  Y  G  M  E  L  I  I  M  I  V  C  T  I  L  Q  T  T  V  A  H  S  P  A  I  N  F
UVA     TTTATGGTTTGGAACTTATTATCATGATTGTTTGTACCATTTTGCAAACCACCGTCGCTCACTCTCCTGCCATCAACTTC
        I  Y  G  L  E  L  I  I  M  I  V  C  T  I  L  Q  T  T  V  A  H  S  P  A  I  N  F

               490       500       510       520       530       540       550       560
        ....|....|....|....|....|....|....|....|....|....|....|....|....|....|....|....|
S288C   GTTGCTGTTTTAACATTCTACCGTATTGTCATGGGTATTGGTATCGGTGGTGACTACCCACTATCTTCTATTATTACTTC
         V  A  V  L  T  F  Y  R  I  V  M  G  I  G  I  G  G  D  Y  P  L  S  S  I  I  T  S
GRF167  GTTGCTGTTTTAACATTCTACCGTATCGTCATGGGTATTGGTATCGGTGGTGACTACCCACTATCTTCTATTATTACTTC
         V  A  V  L  T  F  Y  R  I  V  M  G  I  G  I  G  G  D  Y  P  L  S  S  I  I  T  S
UVA     GTTGCCGTTTTGACCTTCTACCGTATTGTCATGGGTATCGGTATCGGTGGTGACTACCCTCTATCTTCTATTATTACTTC
         V  A  V  L  T  F  Y  R  I  V  M  G  I  G  I  G  G  D  Y  P  L  S  S  I  I  T  S

               570       580       590       600       610       620       630       640
        ....|....|....|....|....|....|....|....|....|....|....|....|....|....|....|....|
S288C   TGAATTTGCCACTACCAAATGGAGAGGTGCCATCATGGGTGCTGTCTTTGCTAACCAAGCTTGGGGTCAAATCTCCGGTG
          E  F  A  T  T  K  W  R  G  A  I  M  G  A  V  F  A  N  Q  A  W  G  Q  I  S  G
GRF167  TGAATTTGCCACTACCAAATGGAGAGGTGCCATCATGGGTGCTGTCTTTGCTAACCAAGCTTGGGGTCAAATTTCCGGTG
          E  F  A  T  T  K  W  R  G  A  I  M  G  A  V  F  A  N  Q  A  W  G  Q  I  S  G
UVA     TGAATTTGCTACTACCAAATGGAGAGGTGCCATCATGGGTGCCGTCTTCGCCAACCAAGCCTGGGGTCAAATCTCAGGTG
          E  F  A  T  T  K  W  R  G  A  I  M  G  A  V  F  A  N  Q  A  W  G  Q  I  S  G

               650       660       670       680       690       700       710       720
        ....|....|....|....|....|....|....|....|....|....|....|....|....|....|....|....|
S288C   GTATCATCGCTCTTATCTTGGTTGCTGCTTACAAGGGCGAACTAGAATACGCAAACTCTGGTGCTGAATGTGATGCTAGA
        G  I  I  A  L  I  L  V  A  A  Y  K  G  E  L  E  Y  A  N  S  G  A  E  C  D  A  R
GRF167  GTATCATCGCTCTTATCTTGGTTGCTGCTTACAAGGGCGAACTAGATTACGCAAACTCTGGTGCTGAATGTGATGCTAGA
        G  I  I  A  L  I  L  V  A  A  Y  K  G  E  L  D  Y  A  N  S  G  A  E  C  D  A  R
UVA     GTATCATCGCCCTTATTTTGGTTGCCGCTTACAAGAATGACTTAAACTATGCTAACTCCGGTGCCGAATGTGACGCCAGA
        G  I  I  A  L  I  L  V  A  A  Y  K  N  D  L  N  Y  A  N  S  G  A  E  C  D  A  R

               730       740       750       760       770       780       790       800
        ....|....|....|....|....|....|....|....|....|....|....|....|....|....|....|....|
```

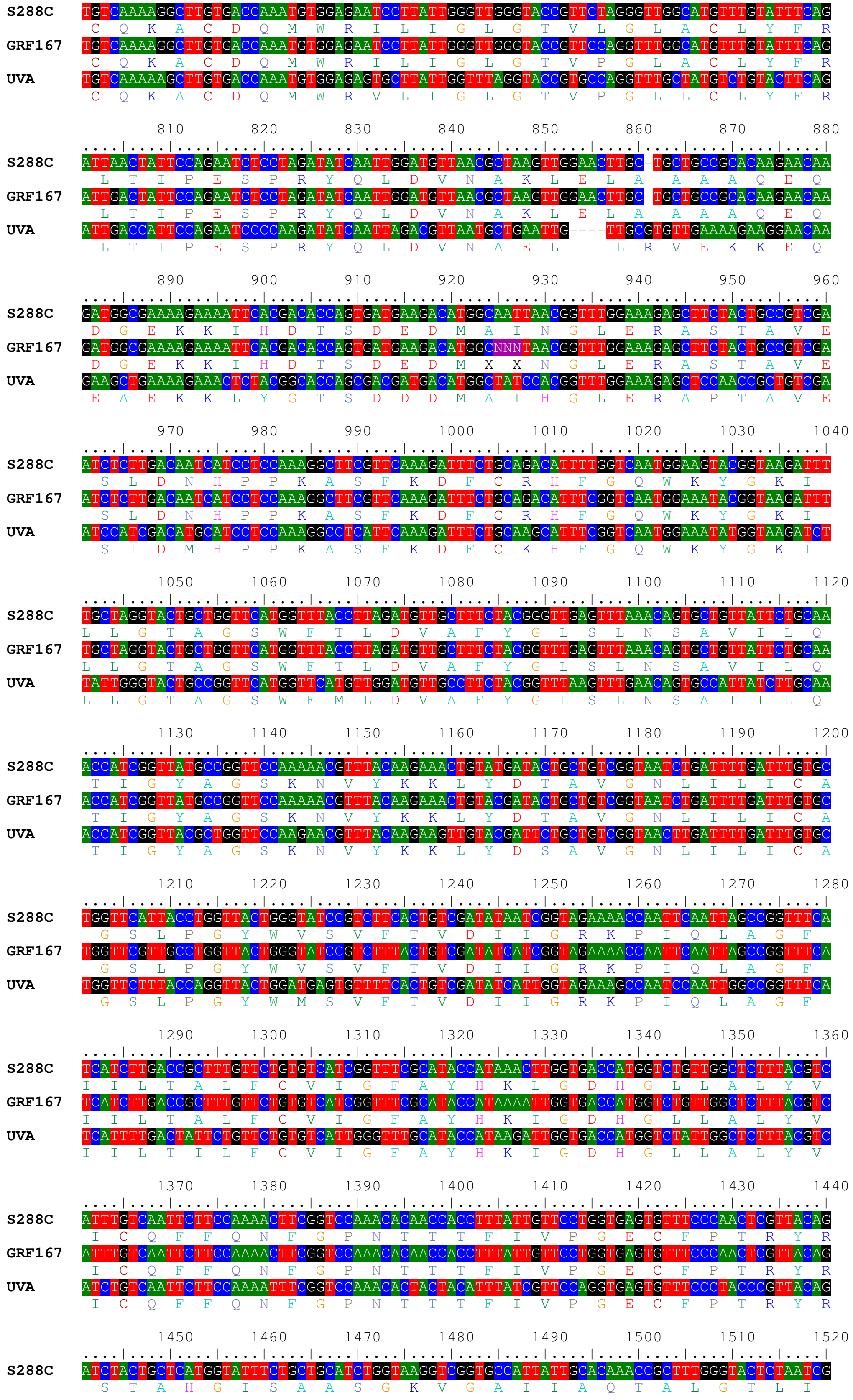
S288C
GRF167
UVA
810 820 830 840 850 860 870 880
890 900 910 920 930 940 950 960
970 980 990 1000 1010 1020 1030 1040
1050 1060 1070 1080 1090 1100 1110 1120
1130 1140 1150 1160 1170 1180 1190 1200
1210 1220 1230 1240 1250 1260 1270 1280
1290 1300 1310 1320 1330 1340 1350 1360
1370 1380 1390 1400 1410 1420 1430 1440
1450 1460 1470 1480 1490 1500 1510 1520

```
GRF167    ATCTACTGCTCATGGTATTTCTGCTGCATCTGGTAAGGTCGGTGCCATTATTGCACAAACCGCTTTGGGTACTCTAATCG
           S  T  A  H  G  I  S  A  A  S  G  K  V  G  A  I  I  A  Q  T  A  L  G  T  L  I 
UVA       ATCTACTGCTCATGGTATTTCTGCCGCATCCGGTAAGATTGGTGCCATTATCGCCCAAACTGCCTTGGGTACTTTGATCA
           S  T  A  H  G  I  S  A  A  S  G  K  I  G  A  I  I  A  Q  T  A  L  G  T  L  I 

                    1530      1540      1550      1560      1570      1580      1590      1600
          ....|....|....|....|....|....|....|....|....|....|....|....|....|....|....|....|
S288C     ACCATAACTGTGCTAGAGACGGTAAGCCAACCAACTGTTGGTTACCTCACGTCATGGAAATTTTCGCCTTATTCATGTTG
          D  H  N  C  A  R  D  G  K  P  T  N  C  W  L  P  H  V  M  E  I  F  A  L  F  M  L 
GRF167    ACCATAACTGTGCTAGAGACGGTAAGCCAACCAACTGTTGGTTACCTCACGTCATGGAAATTTTCGCCTTATTCATGTTG
          D  H  N  C  A  R  D  G  K  P  T  N  C  W  L  P  H  V  M  E  I  F  A  L  F  M  L 
UVA       ACCACAACTGTGCCAAGGACGGTAAGGCCACTAACTGTTGGTTACCTCACGTCATGGAAATCTTTGCCTTGTTTATGTTA
          N  H  N  C  A  K  D  G  K  A  T  N  C  W  L  P  H  V  M  E  I  F  A  L  F  M  L 

                    1610      1620      1630      1640      1650      1660      1670      1680
          ....|....|....|....|....|....|....|....|....|....|....|....|....|....|....|....|
S288C     TTGGGTATCTTCACAACCTTGTTGATCCCAGAAACTAAGAGAAAGACTCTAGAAGAAATTAACGAGCTATACCACGATGA
           L  G  I  F  T  T  L  L  I  P  E  T  K  R  K  T  L  E  E  I  N  E  L  Y  H  D  E
GRF167    TTGGGTATCTTCACAACCTTGTTGATCCCAGAAACTAAGAGAAAGACTCTAGAAGAAATTAACGAGCAATACCACGATGA
           L  G  I  F  T  T  L  L  I  P  E  T  K  R  K  T  L  E  E  I  N  E  Q  Y  H  D  E
UVA       TTAGGTATCTTCACTACTCTGTTGATTCCAGAAACCAAGAGAAAGACCCTGGAAGAAATCAACGAGAAATACCACGATGA
           L  G  I  F  T  T  L  L  I  P  E  T  K  R  K  T  L  E  E  I  N  E  K  Y  H  D  E

                    1690      1700      1710      1720      1730      1740      1750      1760
          ....|....|....|....|....|....|....|....|....|....|....|....|....|....|....|....|
S288C     AATCGATCCTGCTACGCTAAACTTCAGAAACAAGAATAATGACATTGAATCTTCCAGCCCATCTCAACTTCAACATGAAG
            I  D  P  A  T  L  N  F  R  N  K  N  N  D  I  E  S  S  S  P  S  Q  L  Q  H  E  
GRF167    AATCGATCCTGCTACCCTAAACTTCAGAAACAAGAATAATGACATTGAATCTTCCAGCCCATCTCAACTTCAACATGAAG
            I  D  P  A  T  L  N  F  R  N  K  N  N  D  I  E  S  S  S  P  S  Q  L  Q  H  E  
UVA       AATCGACCCTGGTACGCTCAACTACAGAAACAAAATGAACGATGTCGAATCTTCTTCTCCATCTCAAATTCAACATTGAG
            I  D  P  G  T  L  N  Y  R  N  K  M  N  D  V  E  S  S  S  P  S  Q  I  Q  H  *  


          ....|...
S288C     CATAA---
          A  *
GRF167    CATAA---
          A  *
UVA       CTTACTGA
          A  Y  *
```
